# Admission Blood Glucose and 2-Year Mortality After Acute Myocardial Infarction in Patients With Different Glucose Metabolism Status: A Prospective, Nationwide, and Multicenter Registry

**DOI:** 10.3389/fendo.2022.898384

**Published:** 2022-06-15

**Authors:** Kongyong Cui, Rui Fu, Jingang Yang, Haiyan Xu, Dong Yin, Weihua Song, Hongjian Wang, Chenggang Zhu, Lei Feng, Zhifang Wang, Qingsheng Wang, Ye Lu, Kefei Dou, Yuejin Yang

**Affiliations:** ^1^ Cardiometabolic Medicine Center, Department of Cardiology, Fuwai Hospital, National Center for Cardiovascular Diseases, State Key Laboratory of Cardiovascular Disease, Chinese Academy of Medical Sciences and Peking Union Medical College, Beijing, China; ^2^ Coronary Heart Disease Center, Department of Cardiology, Fuwai Hospital, National Center for Cardiovascular Diseases, State Key Laboratory of Cardiovascular Disease, Chinese Academy of Medical Sciences and Peking Union Medical College, Beijing, China; ^3^ Department of Cardiology, Xinxiang Central Hospital, The Fourth Clinical College of Xinxiang Medical University, Xinxiang, China; ^4^ Department of Cardiology, Qinhuangdao First Hospital, Qinhuangdao, China; ^5^ Medical Research & Biometrics Center, Fuwai Hospital, National Center for Cardiovascular Diseases, Chinese Academy of Medical Sciences and Peking Union Medical College, Beijing, China

**Keywords:** stress hyperglycemia, admission blood glucose, acute myocardial infarction, glucose metabolism status, 2-year mortality

## Abstract

**Background:**

The prognostic effect of admission blood glucose (ABG) for patients with acute myocardial infarction (AMI) has not been well validated, especially in patients with diabetes. We performed this study to assess the predictive value of ABG for all-cause mortality in AMI patients with different glucose metabolism status.

**Methods:**

We evaluated a total of 6,892 AMI patients from the prospective, nationwide, multicenter CAMI registry, of which 2,820 had diabetes, 2,011 had pre-diabetes, and 2,061 had normal glucose regulation (NGR). Patients were divided into high ABG and low ABG groups according to the optimal cutoff values of ABG to predict 2-year mortality for patients with diabetes, pre-diabetes and NGR, respectively. The primary endpoint was all-cause mortality.

**Results:**

The optimal cutoff values of ABG for predicting 2-year mortality was 9.0mmol/l, 7.2mmol/l and 6.2mmol/l for patients with diabetes, pre-diabetes and NGR, respectively. Overall, the risk of all-cause mortality in high ABG group was significantly increased compared with that in low ABG group among patients with diabetes (15.2% *vs*. 8.9%; hazard ratio [HR] 1.787, 95% confidence interval [CI] 1.413-2.260; P<0.0001), pre-diabetes (12.1% *vs*. 6.1%; HR 2.069, 95%CI 1.518-2.821; P<0.0001) and NGR (11.8% *vs*. 6.1%; HR 2.009, 95%CI 1.473-2.740; P<0.0001). After the potential confounders were adjusted, high ABG was significantly associated with higher risk of 2-year mortality in patients with diabetes (adjusted HR 1.710, 95%CI 1.327-2.203; P<0.0001), pre-diabetes (adjusted HR 1.731, 95%CI 1.249-2.399; P=0.001) and NGR (adjusted HR 1.529, 95%CI 1.110-2.106; P=0.009). Moreover, adding ABG to the original model led to a slight albeit significant improvement in C-statistic and net reclassification in patients with diabetes and NGR (all P<0.05).

**Conclusions:**

This study is the first to demonstrate a strong positive association between ABG and 2-year mortality in AMI patients with diabetes, pre-diabetes and NGR. ABG should be considered as a useful marker for risk stratification in patients with diabetes and NGR. Further randomized trials are warranted to investigate the effects of blood glucose control on the reduction of long-term mortality according to the corresponding ABG thresholds for different glucose metabolism status.

**Clinical Trial Registration:**

ClinicalTrials.gov, identifier NCT01874691.

## Introduction

Stress hyperglycemia refers to the relative increase of blood glucose level for patients with critical illnesses, including acute myocardial infarction (AMI). Mounting evidence suggested that stress hyperglycemia triggers inflammation and oxidative stress, exacerbates endothelial dysfunction, induces a prothrombotic state, thus leading to coronary flow impairment and increased infarct size in the setting of AMI (1–4). In clinical scenario, admission blood glucose (ABG) has been widely used to define stress hyperglycemia. However, the role of ABG in predicting long-term survival in AMI patients has not been well validated, especially in diabetic patients. Planer et al. reported that high ABG level was an independent predictor of early and late mortality in both AMI patients with and without diabetes mellitus (DM) (5). In contrast, numerous studies showed that ABG level was positively associated with poor outcomes in nondiabetic patients, while its prognostic effect disappeared or attenuated in diabetic patients (6–14). A possible explanation for the controversial results is that the threshold value of ABG has not yet reached a consensus among different studies, varying from 6.7 to 8.0 mmol/l and 10.0 to11.0 mmol/l for patients with and without DM, respectively (6). Some studies even used the same ABG criteria, such as 11.0mmol/l, for all AMI patients and did not differentiate between diabetic and nondiabetic populations (9, 14). Moreover, some studies drew conclusions based on the quintiles, quartiles or tertiles of ABG levels (5, 7, 10–12).

Given that ABG level is subject to both acute stress condition and chronic glycemic levels, it is inappropriate to use a single cutoff value of ABG to define stress hyperglycemia, regardless of different glucose metabolism status. A study with 1,288 AMI patients found that ABG level was an independent predictor of 15-month mortality in nondiabetic patients with the cutoff value of 6.77mmol/l, whereas high ABG was not associated with higher all-cause mortality in diabetic patients with the cutoff value of 14.80mmol/l (13). However, patients were only divided into two subgroups, i.e., diabetic and nondiabetic patients. Pre-DM with mild glucose dysregulation represents a state that is distinct from normal glucose regulation (NGR) and DM. Different from NGR, various metabolic abnormalities already exist before the onset of DM, which are significantly associated with cardiovascular events (15, 16). Nonetheless, only 5~10% of people per year with pre-DM will progress to DM, with the same proportion converting back to NGR (15, 16). In this setting, we used data from China Acute Myocardial Infarction (CAMI) registry to evaluate the predictive value of ABG for 2-year mortality in AMI patients who had DM, pre-DM and NGR with different cutoff values.

## Methods

### Study Design and Population

The CAMI registry is a prospective, nationwide, multicenter and observational study, which is registered in www.Clinicaltrials.gov (NCT01874691). The details of the study design have been described previously (17). From January 2013 to September 2014, eligible AMI patients who were admitted ≤7 days of symptom onset were consecutively enrolled from 108 hospitals throughout China, including those with ST-segment elevation myocardial infarction (STEMI) and non-ST-segment elevation myocardial infarction (NSTEMI). According to the classification of the Third Universal Definition of Myocardial Infarction (MI), only patients with types 1, 2, 3, 4b and 4c of MI were included (18). The members of committees and a complete list of investigators were listed in **Supplementary Tables 1, 2**, respectively. The registry was performed in accordance with the principles of the Declaration of Helsinki and was approved by the Institutional Review Board of each participating hospital. All the participants provided written informed consent before enrollment.

Overall, 26,648 AMI patients were registered during the study period. In this paper, 19,756 patients were excluded because of incomplete or invalid data on ABG or hemoglobin A1c (HbA1c) levels, age, sex, body mass index, admission diagnosis, lipid parameters and in-hospital outcome. Finally, 6,892 AMI patients who met the selection criteria were analyzed. According to the receiver operating characteristic (ROC) curve analysis, the optimal cutoff values of ABG for predicting 2-year mortality in patients with DM, pre-DM and NGR were identified. Then, patients were divided into high ABG group and low ABG group using the respective cutoff values for each subpopulation.

### Data Collection and Definitions

Data were collected, validated, and submitted by trained clinical cardiologists or cardiovascular researchers at each participating site through a secure, password-protected, web-based electronic data capture system. Demographics, cardiovascular risk factors, clinical parameters, laboratory and imaging results, reperfusion strategies, and medications were prospectively recorded using standardized questionnaires, with particular consideration of Chinese patients and hospitals. DM was defined as having a history of diabetes, receiving glucose-lowering therapy before admission, listing DM as a secondary discharge diagnosis in medical records, or having HbA1c ≥ 6.5% at admission (19). Participants who did not have a history of DM or receive glucose-lowering therapy, were not diagnosed with DM but had a HbA1c level ranging from 5.7% to 6.4% at admission were recognized as pre-DM patients (19). NGR patients were those without DM or pre-DM. Definitions of other variables were in compliance with the ACC/AHA Task Force on Clinical Data Standards and the NCDRACTION-GWTG element dictionary (17).

### Follow-Up and Endpoints

We followed up patients at 1, 6, 12, 18 and 24 months after discharge. Data for endpoints were collected from clinical visit or telephone interviews by trained investigators blinded to clinical data. The primary endpoint was 2-year all-cause mortality. Major adverse cardiovascular and cerebrovascular event (MACCE), recurrent MI, stroke, and unplanned revascularization were secondary endpoints. MACCE included all-cause death, recurrent MI, stroke and unplanned revascularization. Recurrent MI was defined according to the Third Universal Definition of MI (18). Unplanned revascularization referred to the revascularization of any coronary lesion by percutaneous coronary intervention (PCI) or coronary artery bypass grafting. Stroke was defined as new focal neurological deficit lasting > 24 hours. It was confirmed by a neurologist based on imaging evidence. Notably, all clinical events must be validated with source documentation.

### Statistical Analysis

Continuous variables were presented as mean ± standard deviation or median (interquartile range) and compared through Student’s t test or Wilcoxon’s rank-sum test, while categorical variables were presented as frequencies (percentages) and compared through Pearson’s chi-square test or Fisher’s exact test, when appropriate. We studied the correlation between ABG and continuous variables through Spearman rank correlation test. Cumulative incidences of 2-year outcomes were estimated with Kaplan-Meier curves, and the differences were assessed with log-rank test. Single-variable and multivariable Cox regression analyses were performed to calculate hazard ratios (HRs) with 95% confidence intervals (CIs). We considered the following variables that were clinically important or statistically significant in the single-variable analysis in the multivariate model: age, sex, body mass index, STEMI, Kiliip class II/III/IV, primary PCI, current smoking, hypertension, previous MI, previous PCI, previous stroke, chronic kidney disease, heart rate, systolic blood pressure, left ventricular ejection fraction (LVEF), HbA1c, triglyceride, low-density lipoprotein cholesterol (LDL-C), and use of statin at discharge.

The cutoff value of ABG was identified by Youden Index using the ROC curve analysis for patients with DM, pre-DM and NGR. The Youden Index is the sum of sensitivity and specificity minus 1, and the optimal cutoff value is the ABG corresponding to the maximum Youden Index. The predictive value of ABG for 2-year mortality was evaluated by area under curve (AUC) with 95% CI (20). In addition, Harrell’s C-statistic and net reclassification improvement (NRI) were compared between different models to assess whether an increased ABG level had incremental prognostic ability (21, 22). In addition, subgroup analysis of the primary endpoint was performed based on important clinical variables, i.e., age, sex, body mass index, diagnosis, primary PCI, anterior MI, and Killip class. In particular, subgroup analysis was also conducted based on the number of diseased vessels and revascularization status for patients who underwent angiography during hospitalization. All statistical analyses were conducted with SPSS version 23.0 (SPSS Inc., Chicago, IL, USA) and R version 3.6.0 (R Foundation for Statistical Computing, Vienna, Austria). A two-sided P value of < 0.05 was considered statistically significant.

## Results

### Baseline Characteristics of Study Population

**Figure 1:**
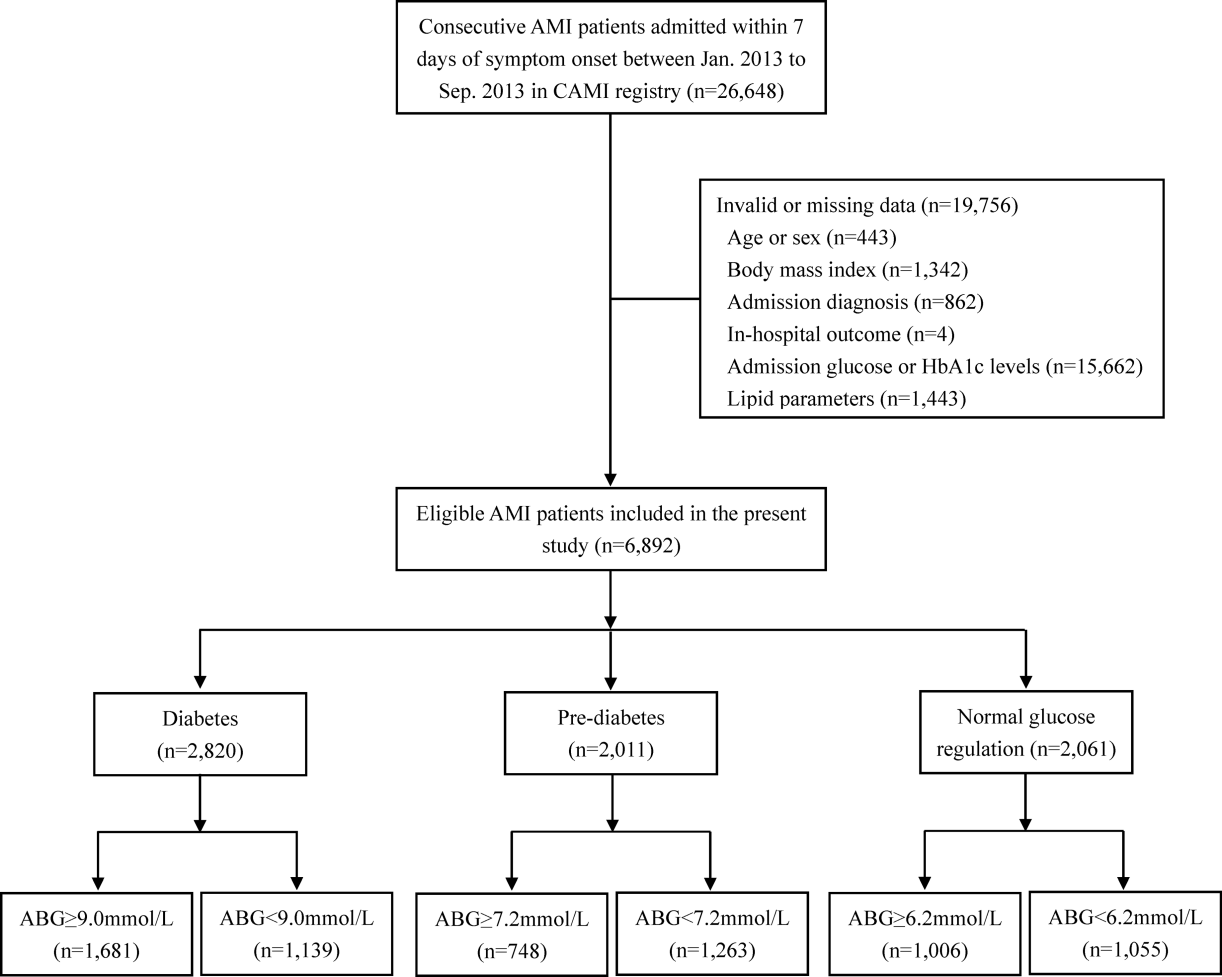
Flow chart of the study. ABG, admission blood glucose; AMI, acute myocardial infarction; CAMI, China Acute Myocardial Infarction.

Among the 6,892 AMI patients, 40.9% (n=2,820) had DM, 29.2% (n=2,011) had pre-DM, and the remaining 29.9% (n=2,061) had NGR **(** **Figure 1** **)**. The median follow-up time was 24.4 (23.3-24.7) months. The optimal cutoff value of ABG for predicting 2-year mortality was 9.0mmol/l, 7.2mmol/l and 6.2mmol/l for patients with DM, pre-DM and NGR, respectively **(** **Supplementary Figure 1** **)**. Of note, the prognostic powers of ABG were similar in patients with DM, pre-DM and NGR (all P < 0.05; **Supplementary Figure 2**).

Accordingly, diabetic patients were divided into ABG ≥ 9.0mmol/l group (n=1,681) and ABG < 9.0mmol/l group (n=1,139). In addition, we used the same method to group patients with pre-DM and NGR, respectively (Pre-DM: ABG ≥ 7.2mmol/l, n=748 and ABG < 7.2mmol/l, n=1,263; NGR: ABG ≥ 6.2mmol/l, n=1,006 and ABG < 6.2mmol/l, n=1,055).

Baseline characteristics of the patients are shown in **Table 1**. Among the three subpopulations, those who had high ABG were less likely to be male, had higher Killip class and white blood cell level but lower LVEF, had more chance to receive primary PCI but less chance to receive aspirin, and had higher in-hospital mortality than those with low ABG (P < 0.05). In addition, among diabetic patients, those who had high ABG were younger, and had higher levels of triglyceride, total cholesterol, LDL-C, HbA1c and hemoglobin than those with low ABG. The pre-DM patients with high ABG were more likely to be diagnosed with STEMI, and their levels of high density lipoprotein cholesterol and HbA1c were higher. The NGR patients with high ABG were older, were more likely to be diagnosed with STEMI, and their levels of total cholesterol, LDL-C, high density lipoprotein cholesterol were higher, but the level of triglyceride were lower.

**Table 1:** Baseline characteristics of the study population according to glucose metabolism status and admission blood glucose levels.

Variable	Diabetes (n=2820)			Pre-diabetes (n=2011)			Normal glucose regulation (n=2061)		
	ABG≥9.0mmol/L (n=1681)	ABG<9.0 mmol/L (n=1139)	P value	ABG≥7.2 mmol/L (n=748)	ABG<7.2 mmol/L (n=1263)	P value	ABG≥6.2 mmol/L (n=1006)	ABG<6.2 mmol/L (n=1055)	P value
Age (year)	62.9 ± 12.0	63.9 ± 11.5	0.025	63.5 ± 12.5	62.8 ± 11.9	0.204	61.4 ± 13.1	59.2 ± 12.9	0.0001
Male	1174 (69.8)	860 (75.5)	0.0009	538 (71.9)	1010 (80.0)	<0.0001	767 (76.2)	890 (84.4)	<0.0001
Body mass index (kg/m^2^)	24.5 ± 3.0	24.8 ± 3.3	0.033	24.3 ± 3.3	24.2 ± 3.2	0.425	24.0 ± 3.1	24.0 ± 3.0	0.913
Current smoking, n (%)	635 (37.8)	477 (41.9)	0.029	338 (45.2)	644 (51.0)	0.012	504 (50.1)	573 (54.3)	0.056
Hypertension, n (%)	1015 (60.4)	745 (65.4)	0.007	422 (56.4)	638 (50.5)	0.010	510 (50.7)	498 (47.2)	0.113
Hyperlipidemia, n (%)	229 (13.6)	148 (13.0)	0.630	100 (13.4)	154 (12.2)	0.445	73 (7.3)	73 (6.9)	0.766
Previous MI, n (%)	141 (8.4)	116 (10.2)	0.106	59 (7.9)	98 (7.8)	0.917	44 (4.4)	52 (4.9)	0.550
Family history of premature CAD, n (%)	79 (4.7)	50 (4.4)	0.699	25 (3.3)	45 (3.6)	0.794	50 (5.0)	39 (3.7)	0.155
Previous PCI, n (%)	78 (4.6)	66 (5.8)	0.174	39 (5.2)	59 (4.7)	0.587	28 (2.8)	29 (2.7)	0.962
Previous CABG, n (%)	14 (0.8)	8 (0.7)	0.698	3 (0.4)	6 (0.5)	1.000	1 (0.1)	3 (0.3)	0.625
Previous stroke, n (%)	152 (9.0)	117 (10.3)	0.277	55 (7.4)	92 (7.3)	0.954	88 (8.7)	80 (7.6)	0.334
Peripheral vascular disease, n (%)	20 (1.2)	8 (0.7)	0.191	1 (0.1)	8 (0.6)	0.167	10 (1.0)	7 (0.7)	0.406
Previous heart failure, n (%)	46 (2.7)	36 (3.2)	0.512	19 (2.5)	20 (1.6)	0.139	19 (1.9)	10 (0.9)	0.070
CKD in treatment, n (%)	34 (2.0)	26 (2.3)	0.640	6 (0.8)	12 (1.0)	0.732	12 (1.2)	6 (0.6)	0.125
COPD, n (%)	23 (1.4)	25 (2.2)	0.099	17 (2.3)	29 (2.3)	0.973	23 (2.3)	20 (1.9)	0.535
STEMI, n (%)	1213 (72.2)	756 (66.4)	0.471	586 (78.3)	910 (72.1)	0.002	798 (79.3)	750 (71.1)	<0.0001
Anterior MI, n (%)	930 (55.3)	621 (54.5)	0.526	420 (56.5)	713 (57.3)	0.707	585 (58.9)	588 (56.3)	0.248
Heart rate (beats/min)	82 ± 19	77 ± 16	<0.0001	79 ± 20	75 ± 15	<0.0001	77 ± 18	76 ± 16	0.125
Systolic blood pressure (mmHg)	131 ± 25	132 ± 24	0.632	127 ± 26	129 ± 24	0.172	127 ± 25	128 ± 23	0.523
LVEF (%)	52.6 ± 10.2	53.7 ± 10.0	0.004	53.0 ± 10.5	54.0 ± 10.7	0.041	54.0 ± 10.1	55.4 ± 10.7	0.002
Killip class II/III/IV, n (%)	490 (29.1)	267 (23.4)	0.001	201 (26.9)	260 (20.6)	0.001	243 (24.2)	166 (15.7)	<0.0001
Primary PCI, n (%)	594 (35.3)	313 (27.5)	<0.0001	326 (43.6)	409 (32.4)	<0.0001	384 (38.2)	303 (28.7)	<0.0001
Coronary angiography during hospitalization, n (%)	1034 (61.5)	717 (62.9)	0.439	477 (63.8)	842 (66.7%)	0.187	629 (62.5)	697 (66.1)	0.093
Laboratory data									
Triglyceride (mmol/L)	1.64 (1.16-2.43)	1.55 (1.11-2.17)	<0.0001	1.32 (0.96-1.84)	1.37 (1.00-1.96)	0.179	1.25 (0.87-1.84)	1.35 (0.96-1.90)	0.031
Total cholesterol (mmol/L)	4.66 (3.95-5.46)	4.41 (3.74-5.12)	<0.0001	4.61 (3.90-5.38)	4.43 (3.80-5.21)	0.087	4.50 (3.87-5.26)	4.31 (3.70-5.01)	<0.0001
LDL-C (mmol/L)	2.78 (2.19-3.40)	2.58 (2.09-3.21)	0.0003	2.80 (2.19-3.43)	2.74 (2.16-3.33)	0.311	2.62 (2.11-3.31)	2.58 (2.08-3.13)	0.013
HDL-C (mmol/L)	1.02 (0.85-1.21)	1.00 (0.84-1.20)	0.222	1.09 (0.91-1.28)	1.03 (0.88-1.23)	0.003	1.10 (0.94-1.31)	1.03 (0.88-1.21)	<0.0001
Admission glucose (mmol/L)	12.78 (10.80-15.60)	7.00 (6.03-7.97)	<0.0001	8.46 (7.70-9.79)	5.83 (5.20-6.50)	<0.0001	7.30 (6.70-8.40)	5.30 (4.80-5.70)	<0.0001
HbA1c (%)	8.30 (7.20-9.80)	7.60 (6.80-9.20)	<0.0001	6.00 (5.80-6.20)	5.90 (5.80-6.10)	<0.0001	5.40 (5.10-5.50)	5.30 (5.10-5.50)	0.357
Serum creatinine (μmol/L)	75.0 (61.4-92.0)	75.8 (63.0-93.0)	0.498	79.1 (63.5-97.0)	76.0 (64.4-90.8)	0.166	74.0 (61.8-89.0)	73.5 (62.3-88.0)	0.336
White blood cell (10^9^/L)	9.80 (7.86-12.58)	8.92 (7.18-11.05)	<0.0001	10.60 (8.15-13.04)	9.22 (7.31-11.70)	<0.0001	10.10 (8.00-12.31)	8.75 (6.90-11.30)	<0.0001
Hemoglobin (g/L)	138 (124-150)	135 (121-147)	0.0006	138 (124-150)	138 (124-149)	0.679	138 (126-151)	141 (128-153)	0.214
Medications at discharge									
Aspirin, n (%)	1482 (88.2)	1050 (92.2)	0.0001	686 (91.7)	1204 (95.3)	0.001	922 (91.7)	993 (94.1)	0.029
Clopidogrel, n (%)	1438 (85.5)	1012 (88.8)	0.010	662 (88.5)	1160 (91.8)	0.014	881 (87.6)	941 (89.2)	0.251
ACEI/ARB, n (%)	1027 (61.1)	731 (64.2)	0.097	494 (66.0)	818 (64.8)	0.561	609 (60.5)	625 (59.2)	0.549
β-blockers, n (%)	1152 (68.5)	812 (71.3)	0.117	562 (75.1)	911 (71.1)	0.140	736 (73.2)	737 (69.9)	0.097
Statins, n (%)	1470 (87.4)	1028 (90.3)	0.020	678 (90.6)	1183 (93.7)	0.014	903 (89.8)	957 (90.7)	0.468
In hospital death, n (%)	102 (6.1)	37 (3.2)	0.0005	44 (5.9)	18 (1.4)	<0.0001	45 (4.5)	22 (2.1)	0.002

ABG, admission blood glucose; ACEI, angiotensin converting enzyme inhibitor; ARB, angiotensin receptor blocker; CABG, coronary artery bypass grafting; CAD, coronary artery disease; CKD, chronic kidney disease; COPD, chronic obstructive pulmonary disease; HbA1c, hemoglobin A1c; HDL-C, high density lipoprotein cholesterol; LDL-C, low-density lipoprotein cholesterol; LVEF, left ventricular ejection fraction; MI, myocardial infarction; NSTEMI, non-ST-segment elevation myocardial infarction; PCI, percutaneous coronary intervention; STEMI, ST-segment elevation myocardial infarction.

The Spearman’s rank correlation analysis showed that ABG was positively correlated with LDL-C and white blood cell among the three subpopulations (P < 0.05). Moreover, ABG was positively correlated with HbA1c in both patients with DM and pre-DM, whereas no significant association was found between the two variables in patients with NGR. Notably, ABG was negatively correlated with age, but positively correlated with triglyceride and hemoglobin in diabetic patients, while the opposite was true in NGR patients **(** **Supplementary Table 3** **)**.

### Association Between ABG Levels and Clinical Outcomes

After a median follow-up of 2 years, a total of 1272 MACCEs were recorded, including 681 deaths, 146 recurrent MIs, 64 strokes, and 500 unplanned revascularizations. As shown in **Supplementary Table 4**, patients who died had significantly higher ABG levels than those who survived at 2 years (P < 0.0001). Additionally, compared with the survivors, the deceased patients were older, were less likely to be male and diagnosed with STEMI, had more prevalent cardiovascular risk factors and comorbidities, and were more likely to have anterior MI, high Killip class, high heart rate but low LVEF. Furthermore, the deceased patients also had significantly higher ABG levels than the survivors in the subpopulations of DM, pre-DM and NGR **(** **Supplementary Table 5** **)**.

**Figure 2:**
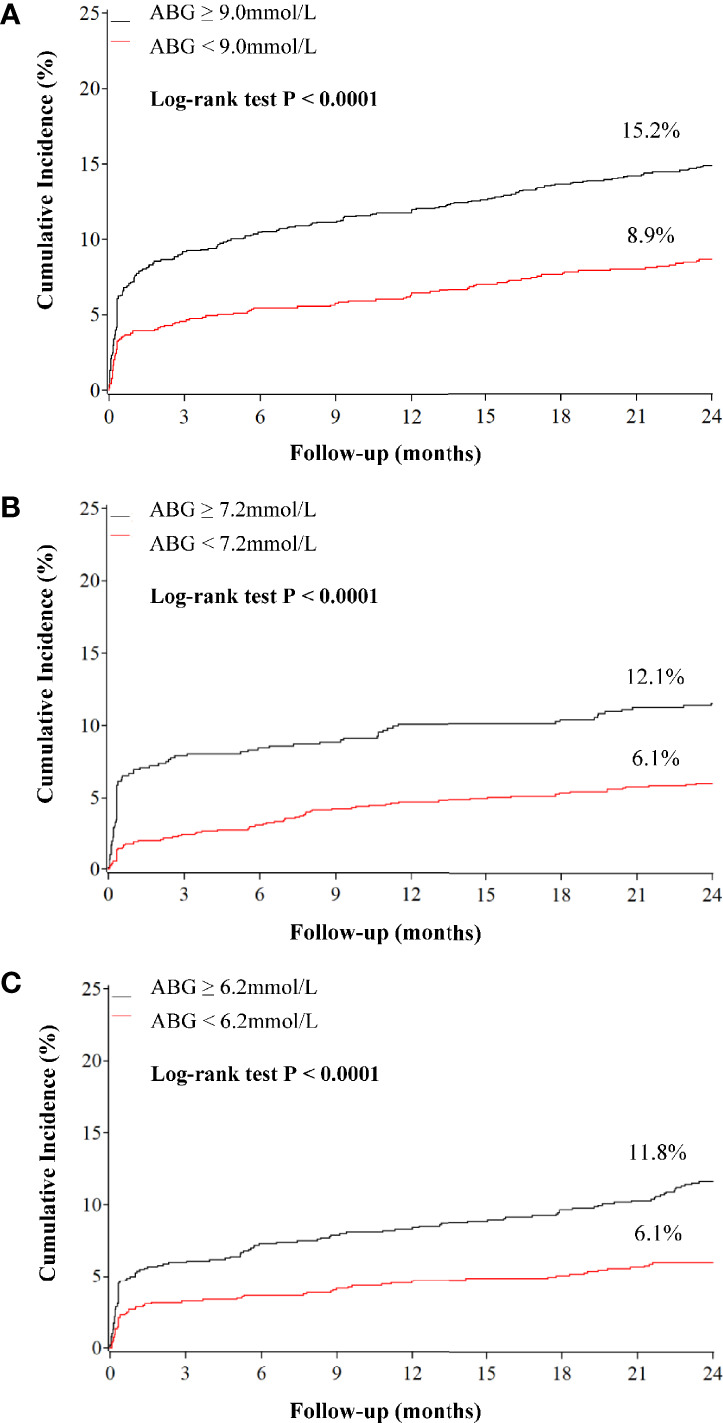
Kaplan–Meier curves for all-cause mortality according to admission blood glucose levels in patients with different glucose metabolism status. **(A)** diabetes; **(B)** pre-diabetes; **(C)** normal glucose regulation. ABG, admission blood glucose.

**Figure 3:**
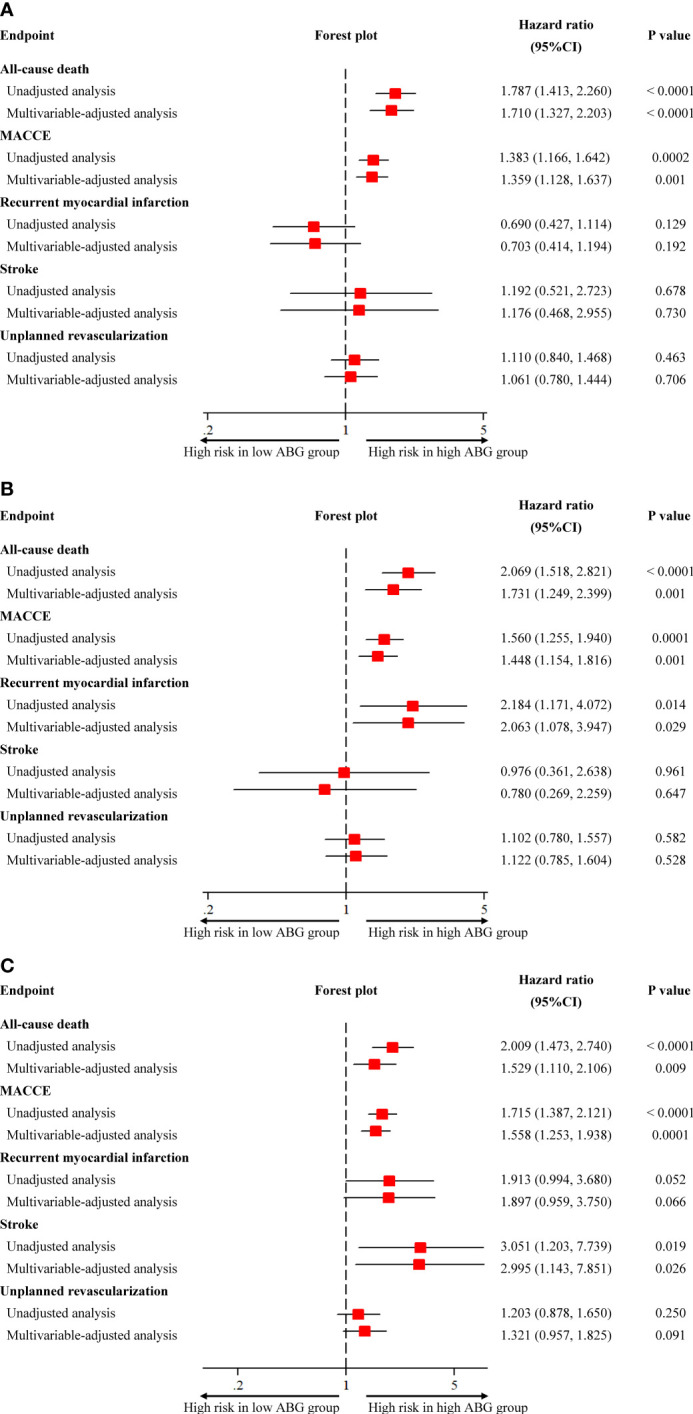
Unadjusted and adjusted association between admission blood glucose and clinical outcomes in patients with different glucose metabolism status. **(A)** diabetes; **(B)** pre-diabetes; **(C)** normal glucose regulation. CI, confidence interval; MACCE, major adverse cardiovascular and cerebrovascular event.

Compared with patients who had low ABG levels, those with high ABG were significantly associated with higher 2-year mortality in patients with DM (HR 1.787, 95%CI 1.413-2.260), pre-DM (HR 2.069, 95%CI 1.518-2.821) and NGR (HR 2.009, 95%CI 1.473-2.740) **(** **Figures 2**, **3** and **Supplementary Table 6** **)**. In addition, the high ABG group also had a higher incidence of MACCE than the low ABG group in the three subpopulations (DM: HR 1.383, 95%CI 1.166-1.642; pre-DM: HR 1.560, 95%CI 1.255-1.940; NGR: HR 1.715, 95%CI 1.387-2.121) **(** **Figure 3** and **Supplementary Figure 3** **;** **Supplementary Table 6** **)**. Furthermore, pre-DM patients with high ABG had a higher risk of recurrent MI (HR 2.184, 95%CI 1.171-4.072) than those with low ABG. The risk of stroke in high ABG group was significantly higher than that in low ABG group for patients with NGR (HR 3.051, 95%CI 1.203-7.739) **(** **Figure 3** and **Supplementary Figure 3** and **Supplementary Table 6** **)**. There were no significant differences between the two groups in terms of unplanned revascularization in the three subpopulations.

After adjusting the potential confounders, high ABG was significantly associated with higher risks of 2-year mortality and MACCE in patients with DM (Death: adjusted HR 1.710, 95%CI 1.327-2.203; MACCE: adjusted HR 1.359, 95%CI 1.128-1.637), pre-DM (Death: adjusted HR 1.731, 95%CI 1.249-2.399; MACCE: adjusted HR 1.448, 95%CI 1.154-1.816) and NGR (Death: adjusted HR 1.529, 95%CI 1.110-2.106; MACCE: adjusted HR 1.558, 95%CI 1.253-1.938). Furthermore, in multivariable Cox regression analysis, high ABG was an independent predictor of recurrent MI for pre-DM patients (adjusted HR 2.063, 95%CI 1.078-3.947) and it was also an independent predictor of stroke for patients with NGR (adjusted HR 2.995, 95%CI 1.143-7.851) **(** **Figure 3** **)**. Other independent predictors for 2-year death were age, Killip class, primary PCI, heart rate, LVEF, and use of statins at discharge in patients with DM, pre-DM and NGR **(** **Table 2** **)**.

**Table 2:** Multivariable analysis for 2-year all-cause mortality in patients with different glucose metabolism status.

Variable	Diabetes		Pre-diabetes		Normal glucose regulation	
	HR (95%CI)	P value	HR (95%CI)	P value	HR (95%CI)	P value
High ABG levels	1.710 (1.327, 2.203)	< 0.0001	1.731(1.249, 2.399)	0.001	1.529 (1.110, 2.106)	0.009
Age (per year)	1.054 (1.042, 1.066)	< 0.0001	1.078 (1.059, 1.097)	< 0.0001	1.054 (1.039, 1.071)	< 0.0001
Male (*vs*. female)	0.717 (0.566, 0.907)	0.006	0.977 (0.676, 1.413)	0.903	0.612 (0.430, 0.869)	0.006
Body mass index (per kg/m^2^)	0.969 (0.933, 1.006)	0.096	1.003 (0.957, 1.051)	0.908	0.938 (0.890, 0.989)	0.017
Current smoker	0.864 (0.649,1.149)	0.315	1.064 (0.740, 1.528)	0.739	0.971 (0.673, 1.400)	0.873
Hypertension	1.098 (0.864, 1.396)	0.445	0.991 (0.700, 1.402)	0.957	1.066 (0.768, 1.479)	0.704
Previous myocardial infarction	1.223(0.880, 1.699)	0.230	1.185 (0.693, 2.025)	0.536	1.380 (0.773, 2.463)	0.276
Previous PCI	0.849 (0.512, 1.408)	0.527	1.189 (0.558, 2.536)	0.654	1.261 (0.564, 2.816)	0.572
Previous stroke	1.277 (0.947, 1.722)	0.110	1.506 (0.935, 2.426)	0.092	1.249 (0.802, 1.947)	0.325
Chronic kidney disease	1.187 (0.723, 1.950)	0.499	2.047 (0.816, 5.133)	0.127	1.682 (0.603, 4.691)	0.321
STEMI (*vs*. NSTEMI)	1.258 (0.988, 1.60)	0.062	1.081 (0.752, 1.554)	0.674	1.192 (0.832, 1.708)	0.339
Heart rate (per beats/min)	1.009 (1.004, 1.014)	0.0004	1.007 (1.000, 1.014)	0.048	1.009 (1.002, 1.015)	0.011
Systolic blood pressure (per mmHg)	0.994 (0.989, 0.998)	0.005	0.991 (0.984, 0.998)	0.008	0.995 (0.990, 1.001)	0.128
LVEF (per 1%)	0.973 (0.963, 0.982)	< 0.0001	0.976 (0.963, 0.989)	0.0002	0.977 (0.964, 0.990)	0.0007
Killip class II/III/IV (*vs*. I)	1.488 (1.178, 1.879)	0.0008	1.451 (1.036, 2.032)	0.030	1.742 (1.249, 2.429)	0.001
Primary PCI	0.442 (0.321, 0.610)	< 0.0001	0.463 (0.304, 0.705)	0.0003	0.452 (0.292, 0.701)	0.0004
Triglyceride (per mmol/L)	0.935 (0.851, 1.027)	0.161	0.933 (0.763, 1.141)	0.501	0.905 (0.732, 1.120)	0.360
LDL-C (per mmol/L)	1.101 (0.986, 1.228)	0.087	1.068 (0.901, 1.267)	0.447	1.014 (0.853, 1.206)	0.871
HbA1c, %	1.012 (0.953, 1.075)	0.700	1.274 (0.612, 2.650)	0.518	0.882 (0.637, 1.223)	0.452
Statins at discharge	0.457 (0.352, 0.594)	< 0.0001	0.313 (0.210, 0.466)	< 0.0001	0.558 (0.381, 0.817)	0.003

ABG, admission blood glucose, CI, confidence interval; HbA1c, hemoglobin A1c; HR, hazard ratio; LDL-C, low-density lipoprotein cholesterol; LVEF, left ventricular ejection fraction; NSTEMI, non-ST-segment elevation myocardial infarction; PCI, percutaneous coronary intervention; STEMI, ST-segment elevation myocardial infarction.

### Risk Prediction for 2-Year Mortality in Different Glucose Metabolism Status

In **Table 3**, C-statistic values for the Cox prediction model consisting of established risk factors were 0.818, 0.827 and 0.837 for patients with DM, pre-DM and NGR, respectively. Adding ABG to the original model, we found a significant improvement in C-statistic in patients with DM (P = 0.034) and NGR (P = 0.012). Furthermore, the addition of ABG to the model resulted in a significant increase in NRI in patients with DM (P = 0.0003) and NGR (P = 0.001). However, there was no significant improvement in C-statistic or NRI when ABG was added to the original model in pre-DM patients.

**Table 3:** Discrimination and reclassification performance of admission blood glucose levels in predicting 2-year mortality.

	C-Statistic (95% CI)	ΔC-statistic (95% CI)	P value	NRI (95% CI)	P value
**Diabetes**					
Established risk factors	0.818 (0.795, 0.842)	Reference		Reference	
Established risk factors+ ABG	0.824 (0.801, 0.847)	0.005 (0.0004, 0.010)	0.034	20.7% (9.5%, 32.0%)	0.0003
**Pre-diabetes**					
Established risk factors	0.827 (0.795, 0.859)	Reference		Reference	
Established risk factors+ ABG	0.829 (0.798, 0.861)	0.002 (-0.002, 0.007)	0.260	7.4% (-8.6%, 23.3%)	0.372
**Normal glucose regulation**					
Established risk factors	0.837 (0.804, 0.870)	Reference		Reference	
Established risk factors+ ABG	0.844 (0.812, 0.876)	0.007 (0.002, 0.012)	0.012	25.2% (9.9%, 40.5%)	0.001

Original model included age, sex, body mass index, ST-elevation myocardial infarction, Kiliip class II/III/IV, primary percutaneous coronary intervention, current smoking, hypertension, previous myocardial infarction, previous percutaneous coronary intervention, previous stroke, chronic kidney disease, heart rate, systolic blood pressure, left ventricular ejection fraction, hemoglobin A1c, triglyceride, low-density lipoprotein cholesterol, and use of statin at discharge. CI, confidence interval; NRI, net reclassification improvement; ABG, admission blood glucose.

### Subgroup Analysis

**Figure 4:**
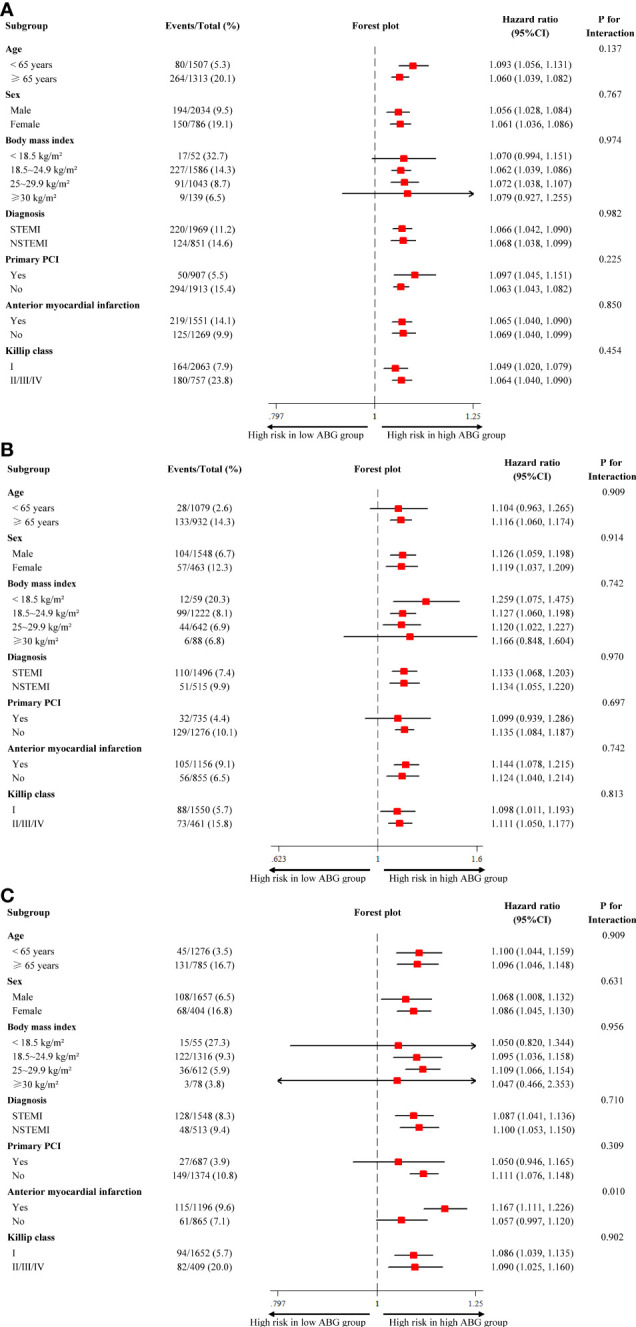
Subgroup analysis for all-cause mortality in patients with different glucose metabolism status. **(A)** diabetes; **(B)** pre-diabetes; **(C)** normal glucose regulation. ABG, admission blood glucose; CI, confidence interval; NSTEMI, non-ST-segment elevation myocardial infarction; PCI, percutaneous coronary intervention; STEMI, ST-segment elevation myocardial infarction.

The results of subgroup analysis were in line with the overall analysis and formal testing for interactions showed that the relative risks of 2-year death between high ABG and low ABG groups were consistent across almost all subgroups among patients with DM, pre-DM and NGR (P _interaction_ > 0.05). However, in the NGR subpopulation, high ABG had a more pronounced effect on the prognosis of patients with anterior MI, whereas there was no significant difference between high ABG and low ABG groups in patients with non-anterior MI (P _interaction_ = 0.010) **(** **Figure 4** **)**. For patients who underwent angiography during hospitalization, no interaction was found between the number of diseased vessels (single-vessel *vs*. multivessel disease) and ABG levels for all-cause mortality in patients with different glucose metabolism status (all P _interaction_ > 0.05; **Supplementary Tables 7–9**). In patients who did not undergo complete revascularization, all-cause mortality of the high ABG group was higher than that of the low ABG group, regardless of glucose metabolism status. However, there was no significant difference between the two groups in patients underwent complete revascularization because of the small sample size **(** **Supplementary Tables 7–9** **)**.

## Discussion

This prospective, nationwide, multicenter, large-scale registry is the first to demonstrate a strong positive association between high ABG and 2-year mortality in AMI patients with DM, pre-DM and NGR. The predictive value of ABG for 2-year mortality was independent of glucose metabolism status. In addition, we identified cutoff values of 9.0mmol/l, 7.2mmol/l and 6.2mmol/l for ABG to discriminate the high-risk subgroups of patients with DM, pre-DM and NGR, respectively. Moreover, C-statistic and NRI analyses further proved that ABG could slightly but significantly improve the predictive value of 2-year mortality in patients with DM and NGR.

Stress hyperglycemia in the setting of AMI is caused by a combination of pancreatic β-cell dysfunction and acute insulin resistance. Bartnik et al. found that plasma proinsulin concentration and the proinsulin/insulin ratio were significantly higher in AMI patients than that in the control group, indicating the presence of β-cell dysfunction in these patients (23). Moreover, sympathetic nervous system activation raises the production of glucagon, catecholamine, cytokine and cortisol that stimulate the glucose release from liver, mobilizes circulating free fatty acids (FFAs) from adipose tissue and contributes to insulin resistance (24–27). Actually, the lack of insulin associated with hyperglycemia may lead to a decrease of glycolytic substrate for cardiac muscle and excessive FFAs. This further exacerbates the reduction in myocardial contractility and increases the risk of pump failure and arrhythmia (28). More importantly, the acute increase of ABG level activates inflammation and oxidative stress, exacerbates endothelial dysfunction and microcirculatory disturbance, induces a prothrombotic state, and then leads to coronary flow impairment, increased infarct size and poor cardiac function, which are closely related to long-term prognosis (1–4). For example, Timmer et al. reported that hyperglycemia was a strong predictor of no reperfusion before primary PCI (odds ratio 2.6, 95%CI 1.5-4.5) (4).

ABG level has been widely used as an indicator of stress hyperglycemia. However, its effect in predicting long-term prognosis in AMI patients has not been well demonstrated, especially in diabetic patients. A secondary analysis of the HORIZONS-AMI trial with 3,602 patients showed that high ABG level was associated with higher 30-day and 3-year mortality in diabetic and nondiabetic patients who underwent primary PCI after STEMI (5). However, many studies showed that the prognostic powers of ABG levels seemed to be different in diabetic and nondiabetic patients, as the ABG was closely associated with poor outcomes in patients without DM, whereas this effect disappeared or attenuated in patients with DM (6–14). One of the most likely reasons is that there is no generally acknowledged cutoff value of ABG levels for diagnosing stress hyperglycemia. For example, some studies used the ABG cutoff values of 6.7~8.0 mmol/l for nondiabetic patients and 10.0~11.0 mmol/l for diabetic patients (6). Some studies even used the same ABG criteria of 10mmol/l or 11.0mmol/l for all AMI patients without distinguishing between diabetic and nondiabetic populations (9, 14). Furthermore, some studies made the conclusions based on the quintiles, quartiles or tertiles of ABG levels (5, 7, 10–12).

ABG level is subject to both acute stress condition and chronic glycemic levels. In this setting, it is not convincing to define stress hyperglycemia by a single cutoff value of ABG, regardless of different glucose metabolism status. Cai et al. found that ABG level was an independent predictor of 15-month all-cause mortality and MACCE in nondiabetic patients with the cutoff value of 6.77mmol/l, whereas high ABG was not associated with high risk of all-cause mortality and MACCE in diabetic patients with the cutoff value of 14.80mmol/l (13). However, there were some limitations in this paper, such as the small sample size and short follow-up time. In addition, patients were only divided into two subgroups, i.e., diabetic and nondiabetic patients. Pre-DM is a high-risk state for the development of diabetes with glucose concentrations or HbA1c levels higher than normal, but lower than DM thresholds. Different from patients with NGR, pre-DM patients already have various metabolic abnormalities, which are associated with cardiovascular events (15, 16). Nonetheless, only 5~10% of people with pre-DM will become diabetic every year, and it can convert back to NGR through lifestyle and drug-based interventions (15, 16, 29). Therefore, studies are warranted to assess the predictive value of ABG for long-term mortality in AMI patients with DM, pre-DM and NGR using the respective cutoff values.

Based on data from the CAMI registry, we obtained the cutoff values (DM: 9.0mmol/l; pre-DM: 7.2mmol/l; and NGR: 6.2mmol/l) for ABG to define stress hyperglycemia and distinguish high-risk patients. Compared with patients with low ABG, those with high ABG had a 1.79-, 2.01-, and 2.00-fold higher risk of 2-year mortality in the subpopulations of DM, pre-DM and NGR, respectively. Furthermore, our results showed that adding ABG to the model with traditional risk factors improved the discrimination of 2-year mortality risk prediction in patients with DM and NGR. Notably, chronic glycemic levels have a significant impact on the ABG levels. For example, some diabetic patients have received optimal glucose-lowering therapy and achieved a good glycemic control, while others have not. Obviously, the latter are more likely to have higher ABG levels. HbA1c indicates average blood glucose levels over the past 2~3 months. Therefore, HbA1c and other 18 variables that may affect the prognosis of patients were included in the Cox proportional hazards regression analysis. Finally, multivariable-adjusted analysis proved the reliability of the results in this paper.

The cutoff values of ABG for AMI patients can help to tailor glucose-lowering treatment according to different diabetic status. Although insulin therapy provides potential benefits to the ischemic myocardium for AMI patients with hyperglycemia, current randomized trials assessing the effect of insulin-based therapy on prognosis failed to demonstrate consistent benefits of insulin-based therapy in this population. As mentioned earlier, stress hyperglycemia represents an epiphenomenon of pancreatic β-cell dysfunction, adrenergic and renin-angiotensin-aldosterone system (RAAS) overactivity, hyperglucagonemia, and the increase of FFAs (23–27, 30, 31). Therefore, in addition to insulin alone, multitargeted therapy is critical for the treatment of stress hyperglycemia. For example, the use of highly β1-selective blockers, RAAS modulators and n3-polyunsaturated fatty acids can counteract adrenergic and RAAS overdrive, and FFAs concentration increase. In addition, glucagon-like peptide-1 receptor agonists and sodium-glucose co-transporters 2 inhibitors can significantly reduce the glucose levels as well as cardiovascular events (30, 32).

In this paper, there are several limitations that cannot be ignored. First, as a secondary analysis of the CAMI registry, the results should be interpreted as hypothesis generating. Although rigorous multivariable-adjusted analysis was conducted, it was difficult to control all the confounding factors. Second, since only subjects with ABG and HbA1c levels were included in this paper, the possibility of selection bias cannot be ruled out. Third, the LVEF levels during follow-up were not routinely collected. Fourth, the duration of diabetes, glucose-lowering therapy, and the adherence to treatment after discharge were not available. Finally, patients with STEMI and NSTEMI in the analysis may contribute to heterogeneity. Nonetheless, the analysis of subgroups found that high ABG was consistently associated with higher 2-year mortality in both cohorts.

## Conclusions

This prospective, multicenter, nationwide registry is the first to demonstrate a strong positive association between ABG and 2-year mortality in AMI patients with DM, pre-DM and NGR. ABG should be considered as a useful marker for risk stratification in patients with DM and NGR. Further randomized trials are warranted to investigate the effects of blood glucose control on the reduction of long-term mortality according to the corresponding ABG thresholds for different glucose metabolism status.

## Data Availability Statement

The original contributions presented in the study are included in the article/**Supplementary Material**. Further inquiries can be directed to the corresponding authors.

## Ethics Statement

The studies involving human participants were reviewed and approved by the ethical committee of Fuwai Hospital, National Center for Cardiovascular Diseases. The patients/participants provided their written informed consent to participate in this study.

## Author Contributions

KD and YY contributed to the study concept and design. KC, RF, JY, HX, DY, WS, HW, CZ, LF, ZW, and QW acquired data. JY, HX, KC, RF, and YL analyzed and interpreted data. YL performed statistical analysis. KC and RF drafted the manuscript. All authors critically revised the manuscript for important intellectual content. All authors contributed to the article and approved the submitted version.

## Funding

This work was supported by CAMS Innovation Fund for Medical Sciences (CIFMS: 2021-I2M-1-008), Beijing Municipal Health Commission-Capital Health Development Research Project (2020-1-4032), CAMS Innovation Fund for Medical Sciences (CIFMS: 2020-I2M-C&T-B-056) and the Twelfth Five-Year Planning Project of the Scientific and Technological Department of China (2011BAI11B02). The sponsor/funder was not involved in the design of the study; the collection, analysis and interpretation of data; writing the report; and did not impose any restrictions regarding the publication of the report.

## Conflict of Interest

The authors declare that the research was conducted in the absence of any commercial or financial relationships that could be construed as a potential conflict of interest.

## Publisher’s Note

All claims expressed in this article are solely those of the authors and do not necessarily represent those of their affiliated organizations, or those of the publisher, the editors and the reviewers. Any product that may be evaluated in this article, or claim that may be made by its manufacturer, is not guaranteed or endorsed by the publisher.
