# Supplementary material for: Admission Blood Glucose and 2-Year Mortality After Acute Myocardial Infarction in Patients With Different Glucose Metabolism Status: A Prospective, Nationwide, and Multicenter Registry
Source: Front Endocrinol (Lausanne). 2022 Jun 15;13:898384. doi: 10.3389/fendo.2022.898384 (PMC9240290; doi:10.3389/fendo.2022.898384)
Supplement: Supplementary file 1 [file DataSheet_1.docx]

**Supplementary materials**

**Admission blood glucose and 2-year mortality after acute myocardial infarction in patients with different glucose metabolism status: A prospective, nationwide, and multicenter registry**

Supplementary Table 1 Members of committees.

Supplementary Table 2 Investigators in the CAMI registry.

Supplementary Table 3 Spearman's correlation analysis between admission blood glucose and other variables in patients with different glucose metabolism status.

Supplementary Table 4 Baseline characteristics of the overall study population stratified by death.

Supplementary Table 5 Baseline characteristics of the study population stratified by death in patients with different glucose metabolism status.

Supplementary Table 6 Two-year outcomes according to glucose metabolism status and admission blood glucose levels.

Supplementary Table 7 Subgroup analysis for all-cause mortality in patients with diabetes who underwent angiography during hospitalization.

Supplementary Table 8 Subgroup analysis for all-cause mortality in patients with pre-diabetes who underwent angiography during hospitalization.

Supplementary Table 9 Subgroup analysis for all-cause mortality in patients with normal glucose regulation who underwent angiography during hospitalization.

Supplementary Figure 1 The receiver operating characteristic curve of admission blood glucose to predict all-cause mortality in patients with different glucose metabolism status.

Supplementary Figure 2 Comparison of the prognostic power of ABG in patients with different glucose metabolism status.

Supplementary Figure 3 Kaplan-Meier curves for secondary outcomes according to admission blood glucose levels in patients with different glucose metabolism status.

**Supplementary Table 1 Members of committees and teams.**

| Principal investigator | Yuejin Yang |
| --- | --- |
| Scientific Committee | Stephen D. Wiviott, Marc S. Sabatine, Eric D. Peterson, Matthew T. Roe, Ying Xian (USA)  Runlin Gao, Zaijia Chen, Weifeng Shen, Wenling Zhu, Luhua Shen, Jilin Chen, Yaling Han, Yude Chen, Shuigao Jin, Dongfeng Gu, Manlu Zhu (China) |
| Data Monitoring Committee | Shubin Qiao, Bo Xu |
| Executive and Steering Committee | Ruiyan Zhang, Xiaozeng Wang, Weimin Li, Daowen Wang, Jiyan Chen, Xianghua Fu, Jianan Wang, Zheng Wan, Chuanyu Gao, Hongwei Li, Yitong Ma, Yang Zheng, lianqun Cui, Bao Li, Zuyi Yuan, Yin Liu, Shaobin Jia, Biao Xu, Lianglong Chen, Shenhua Zhou, Xiaoshu Cheng, Lang Li, Tianhe Yang, Yongjian Wu, Yuan Wu, Hongbin Yan, Jinqin Yuan, Shijie You, Min Yao, Fengying Chen, Likun Ma, Tao Guo, Bin Li, Gesang Luobu, Wei Li |
| Clinical Support Group | Xiaojin Gao, Xuan Zhang, Yunqing Ye, Qiuting Dong, Rui Fu, Xinxin Yan, Hui Sun, Peiyuan He |
| Project managers | Rongfu Li, Hong Wang, Chen Jin |
| Website and EDC Team | Wei Zhao, Jing Yuan, Ting Zhou, Yue Sun, Hui Li, Lihua Zhang |
| Data Management Team | Yi Sun, Xiaomeng Li, Juan Li |
| Monitor Team | Xuan Jia, Xiaoru Cheng, Guofang Qiao, Jing Li, Xin Huang |
| Statistics Team | Yang Wang, Xinran Tang |

**Supplementary Table 2 Investigators in the CAMI registry.**

| ID | Hospital | Province/  Municipality | City | Investigator |
| --- | --- | --- | --- | --- |
| 1 | Fuwai Hospital | Beijing | Beijing | Yuan Wu |
| 2 | Beijing Friendship Hospital | Beijing | Beijing | Hongwei Li |
| 3 | Beijing Tongren Hospital | Beijing | Beijing | Changlin Lu |
| 4 | Beijing Daxing Hospital | Beijing | Daxing | Shujun Cao |
| 5 | Beijing Mentougou Hospital | Beijing | Mentougou | Dezhao Wang |
| 6 | Beijing Pinggu Hospital | Beijing | Pinggu | Guanglin Wei |
| 7 | Beijing Yanqing Hospital | Beijing | Yanqing | Jianbing Wang |
| 8 | Shanghai Jiaotong University Ruijin Hospital | Shanghai | Shanghai | Ruiyan Zhang |
| 9 | Shanghai 10th Hospital | Shanghai | Shanghai | Yawei Xu |
| 10 | Shanghai Fengxian Hospital | Shanghai | Fengxian | Zengyong Qiao |
| 11 | Tianjin Medical School General Hospital | Tianjin | Tianjin | Zheng Wan |
| 12 | Tianjin Baodi Hospital | Tianjin | Baodi | Yanjun Cao |
| 13 | Chongqing Medical School 2nd Hospital | Chongqing | Chongqing | Yaohui Yin |
| 14 | Harbin Medical School 1st Affiliated Hospital | Heilongjiang | Harbin | Weimin Li |
| 15 | Qiqihar 1st Hospital | Heilongjiang | Qiqihar | Shuqing Wang |
| 16 | Tailai Hospital | Heilongjiang | Tailai | Gang Ma |
| 17 | Suihua 1st Hospital | Heilongjiang | Suihua | Yongchen Cai |
| 18 | Jilin University 1st Hospital | Jilin | Changchun | Yang Zheng |
| 19 | Tonghua Central Hospital | Jilin | Tonghua | Xuxia Zhang |
| 20 | Huinan County Hospital | Jilin | Huinan | Hongyan Guo |
| 21 | Shenyang Northern Hospital | Liaoning | Shenyang | Xiaozeng Wang |
| 22 | Fushun Central Hospital | Liaoning | Fushun | Ling Sun |
| 23 | Xiuyan County Hospital | Liaoning | Xiuyan | Jianhua Wu |
| 24 | Neimonggu Medical College 1st Affiliated Hospital | Inner Mongolia | Hohhot | Fengying Chen |
| 25 | Chifeng Hospital | Inner Mongolia | Chifeng | Ronghai Man |
| 26 | Aohan Hospital | Inner Mongolia | Aohan | Yanjie Li |
| 27 | Hebei Medcial School 2nd Affiliated Hospital | Hebei | Shijiazhuang | Xianghua Fu |
| 28 | Qinhuangdao 1st Hospital | Hebei | Qinhuangdao | Qingshen Wang |
| 29 | Qinhuangdao 2nd Hospital | Hebei | Changli | Liying Zhang |
| 30 | North-China Oil-administration General Hospital | Hebei | Renqiu | Xiaoli Gao |
| 31 | Changzhou Hospital | Hebei | Changzhou | Yali Hu |
| 32 | Hengshui Hardison Hospital | Hebei | Hengshui | Qun Zheng |
| 33 | Shanxi Cardiovascular Hospital | Shanxi | Taiyuan | Bao Li |
| 34 | Changzhi Hospital | Shanxi | Changzhi | Yuping zhang |
| 35 | Tunliu Hospital | Shanxi | Tunliu | Yaohong Dong |
| 36 | Henan Provincial Hospital | Henan | Zhengzhou | Chuanyu Gao |
| 37 | Linzhou Hospital | Henan | Linzhou | Zhoushun Qin |
| 38 | Changyuan Hospital | Henan | Changyuan | Guorui Hou |
| 39 | Xinxiang Central Hospital | Henan | Xinxiang | Lingling Liu |
| 40 | Yanjin Hospital | Henan | Yanjin | Shifeng Ren |
| 41 | Ye County hospital | Henan | Ye County | Dezhou wang |
| 42 | Pindingshan 2nd Hospital | Henan | Pindingshan | Xianting Luan |
| 43 | Anyang Prefecture Hospital | Henan | Anyang | Hui Liu |
| 44 | Puyang People’s Hospital | Henan | Puyang | Liping Ma |
| 45 | Xi’an Jiaotong University 1st Hospital | Shan’xi | Xi’an | Zuyi Yuan |
| 46 | Weinan Central Hospital | Shan’xi | Weinan | Junnong Li |
| 47 | Weinan Central Hospital | Shan’xi | Weinan | Junnong Li |
| 48 | Jiuquan Hospital | Gansu | Jiuquan | Yaofeng Yuan |
| 49 | Jinta Hospital | Gansu | Jinta | Huide Liu |
| 50 | Ningxia Medical College General Hospital | Ningxia | Yinchuan | Shaobin jia |
| 51 | Wuzhong Hospital | Ningxia | Wuzhong | Xianghong Luo |
| 52 | Qinghai University Affiliated Hospital | Qinghai | Xining | Yin Liu |
| 53 | Qinhai Cardiovascular Hospital | Qinhai | Xining | Pinfa Liu |
| 54 | Xining 1st Hospital | Qinghai | Xining | Xianning Zhao |
| 55 | Hainan Prefectural Hospital of Qinghai | Qinghai | Gonghe | Bao Ma |
| 56 | Xinjiang Medical College 1st Affiliated Hospital | Xinjiang | Urumchi | Yitong Ma |
| 57 | Changji Hospital | Xinjiang | Changji | Mao Wang |
| 58 | Fukang Hospital | Xinjiang | Fukang | Shiming Gao |
| 59 | Urumchi Friendship Hospital | Xinjiang | Urumchi | Hang Lu |
| 60 | Shandong Provincial Hospital | Shandong | Jinan | Lianqun Cui |
| 61 | Taian Central Hospital | Shandong | Taian | Huanyi Zhang |
| 62 | Xintai Hospital | Shandong | Xintai | Hongyan Zhang |
| 63 | Nanjing University Gulou Hospital | Jiangsu | Nanjin | Biao Xu |
| 64 | Jiangsu North Hospital | Jiangsu | Yangzhou | Shenghu He |
| 65 | Xuzhou 1st Central Hospital | Jiangsu | Xuzhou | Qiang Fu |
| 66 | Jiangyan Hospital | Jiangsu | Jiangyan | Shihai Shen |
| 67 | Anhui Provincial Hospital | Anhui | Hefei | Likun Ma |
| 68 | Fuyang Hospital | Anhui | Fuyang | Bin Ning |
| 69 | Taihe Hospital | Anhui | Taihe | Jili Fan |
| 70 | Zhejiang University 2nd Affiliated Hospital | Zhejiang | Hangzhou | Yong Sun |
| 71 | Taizhou Enze medical Center | Zhejiang | Taizhou | Lijiang tang |
| 72 | Taizhou Hospital | Zhejiang | Linhai | Danlei Xu |
| 73 | Fujian Medical College Union Hospital | Fujian | Fuzhou | Lianglong Chen |
| 74 | Xiamen Heart Center | Fujian | Xiamen | Yan Wang |
| 75 | Fuqing Hospital | Fujian | Fuqing | Ping chen |
| 76 | Longyan 1st Hospital | Fujian | Longyan | Kaihong Chen |
| 77 | Wuhan Tongji Hospital | Hubei | Wuhan | Daowen wang |
| 78 | Jinzhou 1st Hospital | Hubei | Jinzhou | Shuixian peng |
| 79 | Tianmen 1st Hospital | Hubei | Tianmen | Shuping Wan |
| 80 | Gong’an Hospital | Hubei | Gongan | Laxi Zhang |
| 81 | Central South University Xiangya 2nd Hospital | Hunan | Changsha | Shenhua Zhou |
| 82 | Xiangtan Central Hospital | Hunan | Xiangtan | Jianping Zeng |
| 83 | Xiangxiang Hospital | Hunan | Xiangxiang | Chonglun Zhou |
| 84 | Ya’an Hospital | Sichuan | Ya’an | Haibo zhang |
| 85 | Zigong 1st Hospital | Sichuan | Zigong | Dechao Zhong |
| 86 | Danleng County Hospital | Sichuan | Danleng | Yuquan Xiao |
| 87 | Guangxi Medical College 1st Affiliated Hospital | Guangxi | Nanning | Lang Li |
| 88 | Beihai Hospital | Guangxi | Beihai | Hai Zhu |
| 89 | Hepu Hospital | Guangxi | Hepu | Meisheng Lai |
| 90 | Nanchang University 2nd Affiliated Hospital | Jiangxi | Nanchang | Xiaoshu Cheng |
| 91 | Pingxiang Hospital | Jiangxi | Pingxiang | Junming Ye |
| 92 | Shangli Hospital | Jiangxi | Shangli | Qishou Liu |
| 93 | Guizhou Cardiovascular Hospital | Guizhou | Guiyang | Tianhe Yang |
| 94 | Zunyi 1st Hospital | Guizhou | Zunyi | Zhengqiang Yuan |
| 95 | Honghuagang Hospital | Guizhou | Honghuagang | Chengyuan Zhao |
| 96 | Pan County Hospital | Guizhou | Pan | Xianwen Jiang |
| 97 | Guangdong Provincial Hospital | Guangdong | Guangzhou | Jiyan Chen |
| 98 | Guangzhou Traditional Chinese Medical College 1st Affiliated Hospital | Guangdong | Guangzhou | Wei Wu |
| 99 | Jiangmen Hospital | Guangdong | Jiangmen | Gaoxing Zhang |
| 100 | Heshan Hospital | Guangdong | Heshan | Haiyuan Mai |
| 101 | Kunming Medical College 1st Affiliated Hospital | Yunnan | Kunming | Tao Guo |
| 102 | Yunnan St. John’s Hospital | Yunnan | Kunming | Yi Li |
| 103 | Chuxiong People’s Hosptal | Yunnan | Chuxiong | Xiaoming Liu |
| 104 | Yao’an Hospital | Yunnan | Yao’an | Jinlong Xu |
| 105 | Tibet People’s Hospital | Tibet | Lhasa | Gesang Luobu |
| 106 | Hainan Provincial Hospital | Hainan | Haikou | Bin Li |
| 107 | Sanya Hospital | Hainan | Sanya | Tiansong Wang |
| 108 | Wenchang Hospital | Hainan | Wenchang | Dong Wang |

**Supplementary Table 3 Spearman's correlation analysis between admission blood glucose and other variables in patients with different glucose metabolism status.**

| Variable | Diabetes | | Pre-diabetes | | Normal glucose regulation | |
| --- | --- | --- | --- | --- | --- | --- |
|  | Correlation coefficient (95%CI) | P value | Correlation coefficient (95%CI) | P value | Correlation coefficient (95%CI) | P value |
| Age | -0.054 (-0.090, -0.017) | **0.004** | 0.041 (-0.003, 0.085) | 0.065 | 0.100 (0.057, 0.143) | **< 0.0001** |
| Body mass index | -0.033 (-0.070, 0.004) | 0.079 | 0.026 (-0.017, 0.070) | 0.235 | 0.009 (-0.034, 0.052) | 0.683 |
| Triglyceride | 0.068 (0.031, 0.104) | **0.0003** | -0.018 (-0.061, 0.026) | 0.427 | -0.082 (-0.125,-0.039) | **0.0002** |
| LDL-C | 0.102 (0.065, 0.138) | **< 0.0001** | 0.049 (0.005, 0.092) | **0.028** | 0.050 (0.007, 0.093) | **0.024** |
| HDL-C | 0.029 (-0.008, 0.066) | 0.121 | 0.088 (0.044, 0.131) | **0.0001** | 0.143( 0.101, 0.185) | **< 0.0001** |
| HbA1c | 0.513 (0.485, 0.539) | **< 0.0001** | 0.148 (0.105, 0.191) | **< 0.0001** | 0.039 (-0.004, 0.082) | 0.074 |
| Serum creatinine | -0.009 (-0.046, 0.028) | 0.630 | 0.050 (0.006, 0.093) | **0.026** | -0.004 (-0.047, 0.039) | 0.844 |
| White blood cell | 0.191 (0.155, 0.226) | **< 0.0001** | 0.207 (0.165, 0.248) | **< 0.0001** | 0.185 (0.143, 0.226) | **< 0.0001** |
| Hemoglobin | 0.099 (0.062, 0.135) | **< 0.0001** | 0.028 (-0.016, 0.071) | 0.213 | -0.059 (-0.102,-0.016) | **0.008** |

CI, confidence interval; HbA1c, hemoglobin A1c; HDL-C, high density lipoprotein cholesterol; LDL-C, low-density lipoprotein cholesterol.

**Supplementary Table 4 Baseline characteristics of the overall study population stratified by death**.

| Variable | Death (n=681) | No death (n=6211) | P value | |
| --- | --- | --- | --- | --- |
| Age (year) | 72.0±10.4 | 61.3±12.1 | **<0.0001** | |
| Male | 406 (59.6) | 4833 (77.8) | **<0.0001** | |
| Body mass index (kg/m^2^) | 23.4±3.1 | 24.4±3.1 | **<0.0001** | |
| Current smoking, n (%) | 188 (27.6) | 2983 (48.0) | **<0.0001** | |
| Hypertension, n (%) | 421 (61.8) | 3407 (54.9) | **0.0005** | |
| Hyperlipidemia, n (%) | 56 (8.2) | 721 (11.6) | **0.006** | |
| Previous MI, n (%) | 82 (12.0) | 428 (6.9) | **<0.0001** | |
| Family history of premature CAD, n (%) | 12 (1.8) | 276 (4.4) | **0.0002** | |
| Previous PCI, n (%) | 33 (4.8) | 266 (4.3) | 0.500 | |
| Previous CABG, n (%) | 9 (1.3) | 26 (0.4) | **0.006** | |
| Previous stroke, n (%) | 99 (14.5) | 485 (7.8) | **<0.0001** | |
| Peripheral vascular disease, n (%) | 12 (1.8) | 42 (0.7) | **0.0008** | |
| Previous heart failure, n (%) | 55 (8.1) | 95 (1.5) | **<0.0001** | |
| CKD in treatment, n (%) | 29 (4.3) | 67 (1.1) | **<0.0001** | |
| COPD, n (%) | 40 (5.9) | 97 (1.6) | **<0.0001** | |
| STEMI, n (%) | 458 (67.3) | 4555 (73.3) | **0.0009** | |
| Anterior MI, n (%) | 439 (64.5) | 3464 (55.8) | **<0.0001** | |
| Heart rate (beats/min) | 85±22 | 77±17 | **<0.0001** | |
| Systolic blood pressure (mmHg) | 126±27 | 130±24 | **0.003** | |
| LVEF (%) | 48.7±10.9 | 54.3±10.2 | **<0.0001** | |
| Killip class II/III/IV, n (%) | 335 (49.2) | 1292 (20.8) | **<0.0001** | |
| Primary PCI, n (%) | 109 (16.0) | 2220 (35.7) | **<0.0001** | |
| Coronary angiography during hospitalization, n (%) | 200 (29.4) | 4196 (67.6) | **<0.0001** | |
| Laboratory data |  |  |  | |
| Triglyceride (mmol/L) | 1.22 (0.92-1.71) | 1.46 (1.03-2.10) | **<0.0001** | |
| Total cholesterol (mmol/L) | 4.45 (3.70-5.25) | 4.50 (3.84-5.27) | 0.676 | |
| LDL-C (mmol/L) | 2.65 (2.05-3.35) | 2.68 (2.14-3.31) | 0.297 | |
| HDL-C (mmol/L) | 1.08 (0.90-1.30) | 1.03 (0.87-1.23) | **0.002** | |
| Admission glucose (mmol/L) | 8.47 (6.45-12.34) | 7.12 (5.79-9.73) | **<0.0001** | |
| HbA1c (%) | 6.30 (5.60-7.50) | 6.00 (5.50-7.10) | **0.0001** | |
| Serum creatinine (μmol/L) | 86.0 (69.0-116.0) | 74.6 (62.0-89.3) | **<0.0001** | |
| White blood cell (10^9^/L) | 10.20 (8.00-13.00) | 9.50 (7.49-11.90) | **<0.0001** | |
| Hemoglobin (g/L) | 128 (113-141) | 139 (126-151) | **<0.0001** | |
| Medications at discharge |  | | |  |
| Aspirin, n (%) | 534 (78.4) | 5803 (93.4) | **<0.0001** | |
| Clopidogrel, n (%) | 531 (78.0) | 5563 (89.6) | **<0.0001** | |
| ACEI/ARB, n (%) | 409 (60.1) | 3895 (62.7) | 0.176 | |
| β-blockers, n (%) | 416 (61.1) | 4494 (72.4) | **<0.0001** | |
| Statins, n (%) | 537 (78.9) | 5682 (91.5) | **<0.0001** | |

ACEI, angiotensin converting enzyme inhibitor; ARB, angiotensin receptor blocker; CABG, coronary artery bypass grafting; CAD, coronary artery disease; CKD, chronic kidney disease; COPD, chronic obstructive pulmonary disease; HbA1c, hemoglobin A1c; HDL-C, high density lipoprotein cholesterol; LDL-C, low-density lipoprotein cholesterol; LVEF, left ventricular ejection fraction; MI, myocardial infarction; PCI, percutaneous coronary intervention; STEMI, ST-segment elevation myocardial infarction.

**Supplementary Table 5 Baseline characteristics of the study population stratified by death in patients with different glucose metabolism status**.

| Variable | Diabetes (n=2820) | | | Pre-diabetes (n=2011) | | | Normal glucose regulation (n=2061) | | |
| --- | --- | --- | --- | --- | --- | --- | --- | --- | --- |
|  | Death (n=344) | No death (n=2476) | P value | Death (n=161) | No death (n=1850) | P value | Death (n=176) | No death (n=1885) | P value |
| Age (year) | 71.7±10.2 | 62.2±11.6 | **<0.0001** | 73.0±9.6 | 62.2±11.9 | **<0.0001** | 71.6±11.5 | 59.2±12.7 | **<0.0001** |
| Male | 194 (56.4) | 1840 (74.3) | **<0.0001** | 104 (64.6) | 1444 (78.1) | **0.0002** | 68 (38.6) | 336 (17.8) | **<0.0001** |
| Body mass index (kg/m^2^) | 23.6±3.0 | 24.8±3.1 | **<0.0001** | 23.5±3.4 | 24.3±3.2 | **0.007** | 22.7±3.1 | 24.1±3.0 | **<0.0001** |
| Current smoking, n (%) | 75 (21.8) | 1037 (41.9) | **<0.0001** | 54 (33.5) | 928 (50.2) | **<0.0001** | 59 (33.5) | 1018 (54.0) | **<0.0001** |
| Hypertension, n (%) | 227 (66.0) | 1533 (61.9) | 0.141 | 97 (60.2) | 963 (52.1) | **0.045** | 97 (55.1) | 911 (48.3) | 0.085 |
| Hyperlipidemia, n (%) | 37 (10.8) | 340 (13.7) | 0.119 | 10 (6.2) | 244 (13.2) | **0.005** | 9 (5.1) | 137 (7.3) | 0.266 |
| Previous MI, n (%) | 48 (14.0) | 209 (8.4) | **0.002** | 18 (11.2) | 139 (7.5) | 0.114 | 16 (9.1) | 80 (4.2) | **0.009** |
| Family history of premature CAD, n (%) | 5 (1.5) | 124 (5.0) | **0.0007** | 1 (0.6) | 69 (3.7) | **0.013** | 6 (3.4) | 83 (4.4) | 0.521 |
| Previous PCI, n (%) | 17 (4.9) | 127 (5.1) | 0.882 | 8 (5.0) | 90 (4.9) | 0.953 | 8 (4.5) | 49 (2.6) | 0.145 |
| Previous CABG, n (%) | 5 (1.5) | 17 (0.7) | 0.177 | 2 (1.2) | 7 (0.4) | 0.158 | 2 (1.1) | 2 (0.1) | **0.039** |
| Previous stroke, n (%) | 53 (15.4) | 216 (8.7) | **0.0002** | 21 (13.0) | 126 (6.8) | **0.008** | 25 (14.2) | 143 (7.6) | **0.005** |
| Peripheral vascular disease, n (%) | 11 (3.2) | 17 (0.7) | **0.0002** | 0 (0) | 9 (0.5) | 1.000 | 1 (0.6) | 16 (0.8) | 1.000 |
| Previous heart failure, n (%) | 31 (9.0) | 51 (2.1) | **<0.0001** | 15 (9.3) | 24 (1.3) | **<0.0001** | 9 (5.1) | 20 (1.1) | **0.0004** |
| CKD in treatment, n (%) | 20 (5.8) | 40 (1.6) | **<0.0001** | 5 (3.1) | 13 (0.7) | **0.011** | 4 (2.3) | 14 (0.7) | 0.061 |
| COPD, n (%) | 13 (3.8) | 35 (1.4) | **0.005** | 12 (7.5) | 34 (1.8) | **0.0002** | 15 (8.5) | 28 (1.5) | **<0.0001** |
| STEMI, n (%) | 220 (64.0) | 1749 (70.6) | **0.013** | 110 (68.3) | 1386 (74.9) | 0.072 | 128 (72.7) | 1420 (75.3) | 0.449 |
| Anterior MI, n (%) | 219 (63.7) | 1332 (53.8) | **0.0005** | 105 (65.2) | 1051 (56.8) | **0.037** | 115 (65.3) | 1081 (57.3) | **0.038** |
| Heart rate (beats/min) | 87±23 | 79±17 | **<0.0001** | 82 ±21 | 76 ±16 | **0.0004** | 84 ±22 | 76 ±17 | **<0.0001** |
| Systolic blood pressure (mmHg) | 128±27 | 132±25 | **0.008** | 124±26 | 128±25 | 0.057 | 125±28 | 128±24 | 0.267 |
| LVEF (%) | 48.2±10.2 | 53.7±9.9 | **<0.0001** | 48.5±11.9 | 54.1±10.4 | **<0.0001** | 49.9±11.4 | 55.2±10.2 | **<0.0001** |
| Killip class II/III/IV, n (%) | 180 (52.3) | 577 (23.3) | **<0.0001** | 73 (45.3) | 388 (21.0) | **<0.0001** | 82 (46.6) | 327 (17.3) | **<0.0001** |
| Primary PCI, n (%) | 50 (14.5) | 857 (34.6) | **<0.0001** | 32 (19.9) | 703 (38.0) | **<0.0001** | 27 (15.3) | 660 (35.0) | **<0.0001** |
| Coronary angiography during hospitalization, n (%) | 101 (29.4) | 1650 (66.6) | **<0.0001** | 48 (29.8) | 1271 (68.7) | **<0.0001** | 51 (29.0) | 1275 (67.6) | **<0.0001** |
| Laboratory data |  |  |  |  |  |  |  |  |  |
| Triglyceride (mmol/L) | 1.32 (1.02-1.95) | 1.64 (1.17-2.36) | **<0.0001** | 1.23 (0.92-1.52) | 1.37 (0.98-1.94) | **<0.0001** | 1.02 (0.77-1.39) | 1.32 (0.94-1.90) | **<0.0001** |
| Total cholesterol (mmol/L) | 4.53 (3.75-5.35) | 4.56 (3.88-5.34) | 0.793 | 4.45 (3.72-5.25) | 4.52 (3.82-5.30) | 0.513 | 4.35 (3.50-5.01) | 4.42 (3.80-5.12) | 0.219 |
| LDL-C (mmol/L) | 2.70 (2.09-3.39) | 2.69 (2.15-3.33) | 0.550 | 2.64 (2.13-3.31) | 2.75 (2.17-3.37) | 0.235 | 2.47 (1.82-3.20) | 2.60 (2.12-3.22) | **0.040** |
| HDL-C (mmol/L) | 1.06 (0.87-1.27) | 1.00 (0.84-1.20) | 0.110 | 1.15 (0.92-1.34) | 1.05 (0.88-1.24) | **0.016** | 1.13 (0.92-1.36) | 1.06 (0.91-1.26) | **0.033** |
| Admission glucose (mmol/L) | 11.10 (8.55-16.00) | 10.02 (7.35-13.21) | **<0.0001** | 7.36 (5.94-8.77) | 6.56 (5.60-7.84) | **0.0005** | 6.70 (5.60-8.08) | 6.10 (5.28-7.20) | **<0.0001** |
| HbA1c (%) | 7.50 (6.70-9.20) | 7. 50 (6.70-9.00) | 0.271 | 6.00 (5.80-6.20) | 5.95 (5.80-6.10) | 0.429 | 5.30 (5.10-5.50) | 5.34 (5.10-5.50) | 0.160 |
| Serum creatinine (μmol/L) | 87.0 (72.3-125.3) | 74.0 (61.1-90.0) | **<0.0001** | 88.7 (70.0-117.0) | 76.0 (64.0-91.0) | **<0.0001** | 78.5 (64.0-104.0) | 73.7 (62.0-87.4) | **0.002** |
| White blood cell (10^9^/L) | 10.35 (8.20-13.38) | 9.41 (7.50-11.79) | **<0.0001** | 9.89 (7.90-12.40) | 9.64 (7.60-12.22) | 0.185 | 10.12 (7.55-12.35) | 9.40 (7.30-11.80) | 0.120 |
| Hemoglobin (g/L) | 128 (111-141) | 138 (124-150) | **<0.0001** | 128 (116-141) | 138 (125-150) | **<0.0001** | 129 (117-141) | 140 (129-153) | **<0.0001** |
| Medications at discharge |  | | | | | |  |  |  |
| Aspirin, n (%) | 269 (78.2) | 2263 (91.4) | **<0.0001** | 127 (78.9) | 1763 (95.3) | **<0.0001** | 138 (78.4) | 1777 (94.3) | **<0.0001** |
| Clopidogrel, n (%) | 259 (75.3) | 2191 (88.5) | **<0.0001** | 133 (82.6) | 1689 (91.3) | **0.0009** | 139 (79.0) | 1683 (89.3) | **0.0002** |
| ACEI/ARB, n (%) | 206 (59.9) | 1552 (62.7) | 0.317 | 96 (59.6) | 1216 (65.7) | 0.123 | 107 (60.8) | 1127 (59.8) | 0.794 |
| β-blockers, n (%) | 205 (59.6) | 1759 (71.0) | **<0.0001** | 103 (64.0) | 1370 (74.1) | **0.007** | 108 (61.4) | 1365 (72.4) | **0.003** |
| Statins, n (%) | 267 (77.6) | 2231 (90.1) | **<0.0001** | 128 (79.5) | 1733 (93.7) | **<0.0001** | 142 (80.7) | 1718 (91.1) | **0.0001** |

ACEI, angiotensin converting enzyme inhibitor; ARB, angiotensin receptor blocker; CABG, coronary artery bypass grafting; CAD, coronary artery disease; CKD, chronic kidney disease; COPD, chronic obstructive pulmonary disease; HbA1c, hemoglobin A1c; HDL-C, high density lipoprotein cholesterol; LDL-C, low-density lipoprotein cholesterol; LVEF, left ventricular ejection fraction; MI, myocardial infarction; PCI, percutaneous coronary intervention; STEMI, ST-segment elevation myocardial infarction.

**Supplementary Table 6 Two-year outcomes according to glucose metabolism status and admission blood glucose levels.**

| Clinical endpoint | Diabetes (n=2820) | | P value | Pre-diabetes (n=2011) | | P value | Normal glucose regulation (n=2061) | | | P value |
| --- | --- | --- | --- | --- | --- | --- | --- | --- | --- | --- |
|  | ABG≥9.0mmol/L (n=1681) | ABG<9.0 mmol/L (n=1139) |  | ABG≥7.2 mmol/L (n=748) | ABG<7.2 mmol/L (n=1263) |  | ABG≥6.2 mmol/L (n=1006) | | ABG<6.2 mmol/L (n=1055) |  |
| All-cause death, n (%) | 247 (15.2) | 97 (8.9) | **<0.0001** | 87 (12.1) | 74 (6.1) | **<0.0001** | 115 (11.8) | 61 (6.1) | | **<0.0001** |
| MACCE, n (%) | 389 (23.9) | 198 (18.0) | **0.0002** | 152 (21.2) | 173 (14.2) | **0.0001** | 221 (22.6) | 139 (14.0) | | **<0.0001** |
| Recurrent MI, n (%) | 33 (2.2) | 34 (3.2) | 0.126 | 22 (3.4) | 18 (1.5) | **0.011** | 25 (2.8) | 14 (1.5) | | 0.052 |
| Stroke, n (%) | 15 (1.0) | 9 (0.9) | 0.685 | 6 (0.9) | 11 (0.9) | 0.986 | 17 (1.9) | 6 (0.6) | | **0.013** |
| Unplanned revascularization, n (%) | 128 (8.7) | 81 (7.8) | 0.388 | 52 (8.0) | 84 (7.1) | 0.494 | 82 (9.1) | 73 (7.7) | | 0.266 |

MACCE, major adverse cardiovascular and cerebrovascular event; MI, myocardial infarction.

Supplementary Table 7 Subgroup analysis for all-cause mortality in patients with diabetes who underwent angiography during hospitalization.

| Variables | Events/Total (%) | | HR (95%CI) | P _interaction_ |
| --- | --- | --- | --- | --- |
|  | ABG≥9.0mmol/L  (n=1034) | ABG≥9.0mmol/L  (n=717) |  |  |
| No. diseased vessels |  |  |  | 0.169 |
| Single-vessel disease | 14/238 (5.9) | 3/183 (1.6) | **3.639 (1.046, 12.663)** |  |
| Multivessel disease | 57/796 (7.2) | 27/534 (5.1) | 1.436 (0.908, 2.270) |  |
| Complete revascularization |  |  |  | 0.037 |
| Yes | 2/81 (2.5) | 5/65 (7.7) | 0.316 (0.061, 1.626) |  |
| No | 69/953 (7.2) | 25/652 (3.8) | **1.921 (1.216, 3.035)** |  |

ABG, admission blood glucose; CI, confidence interval; HR, hazard ratio.

Supplementary Table 8 Subgroup analysis for all-cause mortality in patients with pre-diabetes who underwent angiography during hospitalization.

| Variables | Events/Total (%) | | HR (95%CI) | P _interaction_ |
| --- | --- | --- | --- | --- |
|  | ABG≥7.2 mmol/L (n=477) | ABG<7.2 mmol/L (n=842) |  |  |
| No. diseased vessels |  |  |  | 0.602 |
| Single-vessel disease | 5/151 (3.3) | 6/266 (2.3) | 1.489 (0.454, 4.878) |  |
| Multivessel disease | 20/326 (6.1) | 17/576 (3.0) | **2.129 (1.115, 4.065)** |  |
| Complete revascularization |  |  |  | NA |
| Yes | 0/26 (0.0) | 1/52 (1.9) | NA |  |
| No | 25/451 (5.5) | 22/790 (2.8) | **2.037 (1.148, 3.612)** |  |

ABG, admission blood glucose; CI, confidence interval; HR, hazard ratio; NA, not applicable.

Supplementary Table 9 Subgroup analysis for all-cause mortality in patients with normal glucose regulation who underwent angiography during hospitalization.

| Variables | Events/Total (%) | | HR (95%CI) | P _interaction_ |
| --- | --- | --- | --- | --- |
|  | ABG≥6.2 mmol/L (n=629) | ABG<6.2 mmol/L (n=697) |  |  |
| No. diseased vessels |  |  |  | 0.273 |
| Single-vessel disease | 11/197 (5.6) | 3/280 (1.1) | **5.197 (1.450, 18.628)** |  |
| Multivessel disease | 26/432 (6.0) | 11/417 (2.6) | **2.298 (1.136, 4.650)** |  |
| Complete revascularization |  |  |  | 0.741 |
| Yes | 3/48 (6.3) | 1/66 (1.5) | 4.205 (0.437, 40.424) |  |
| No | 34/581 (5.9) | 13/631 (2.1) | **2.846 (1.502, 5.392)** |  |

ABG, admission blood glucose; CI, confidence interval; HR, hazard ratio.

**Supplementary Figure 1 The receiver operating characteristic curve of ABG to predict all-cause mortality in patients with different glucose metabolism status. (A) diabetes; (B) pre-diabetes; (C) normal glucose regulation.** AUC, area under curve; ABG, admission blood glucose.


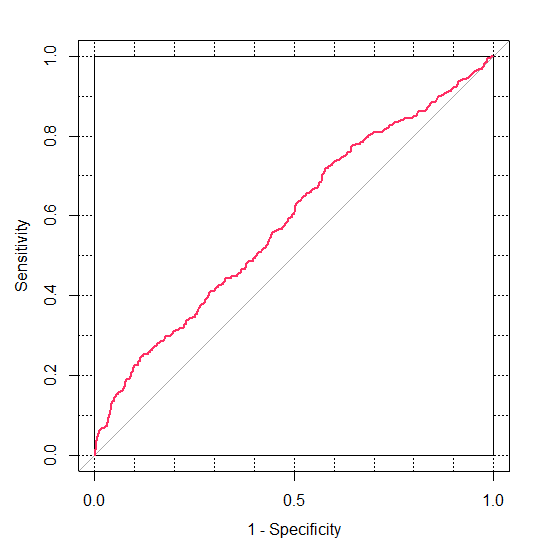


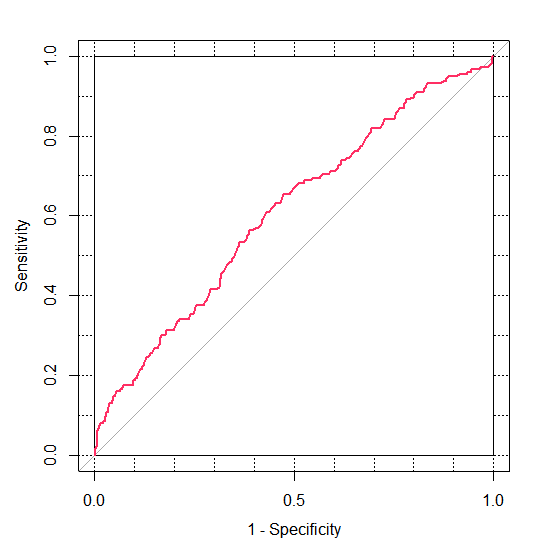
**
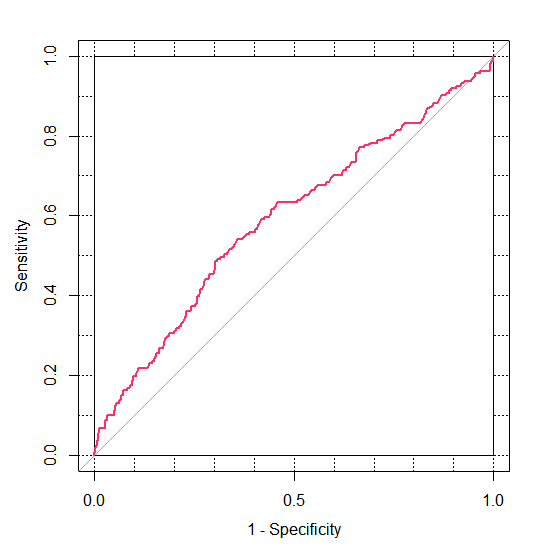
**

AUC: 0.61 (0.56-0.65)

ABG cutoff value: 6.2

Specificity: 65%

Sensitivity: 53%

AUC: 0.59 (0.54-0.64)

ABG cutoff value: 7.2

Specificity: 54%

Sensitivity: 64%

AUC: 0.59 (0.55-0.62)

ABG cutoff value: 9.0

Specificity: 72%

Sensitivity: 42%

**(C)**

**(B)**

**(A)**

**Supplementary Figure 2 Comparison of the prognostic power of ABG in patients with different glucose metabolism status.** AUC, area under curve; ABG, admission blood glucose.

**
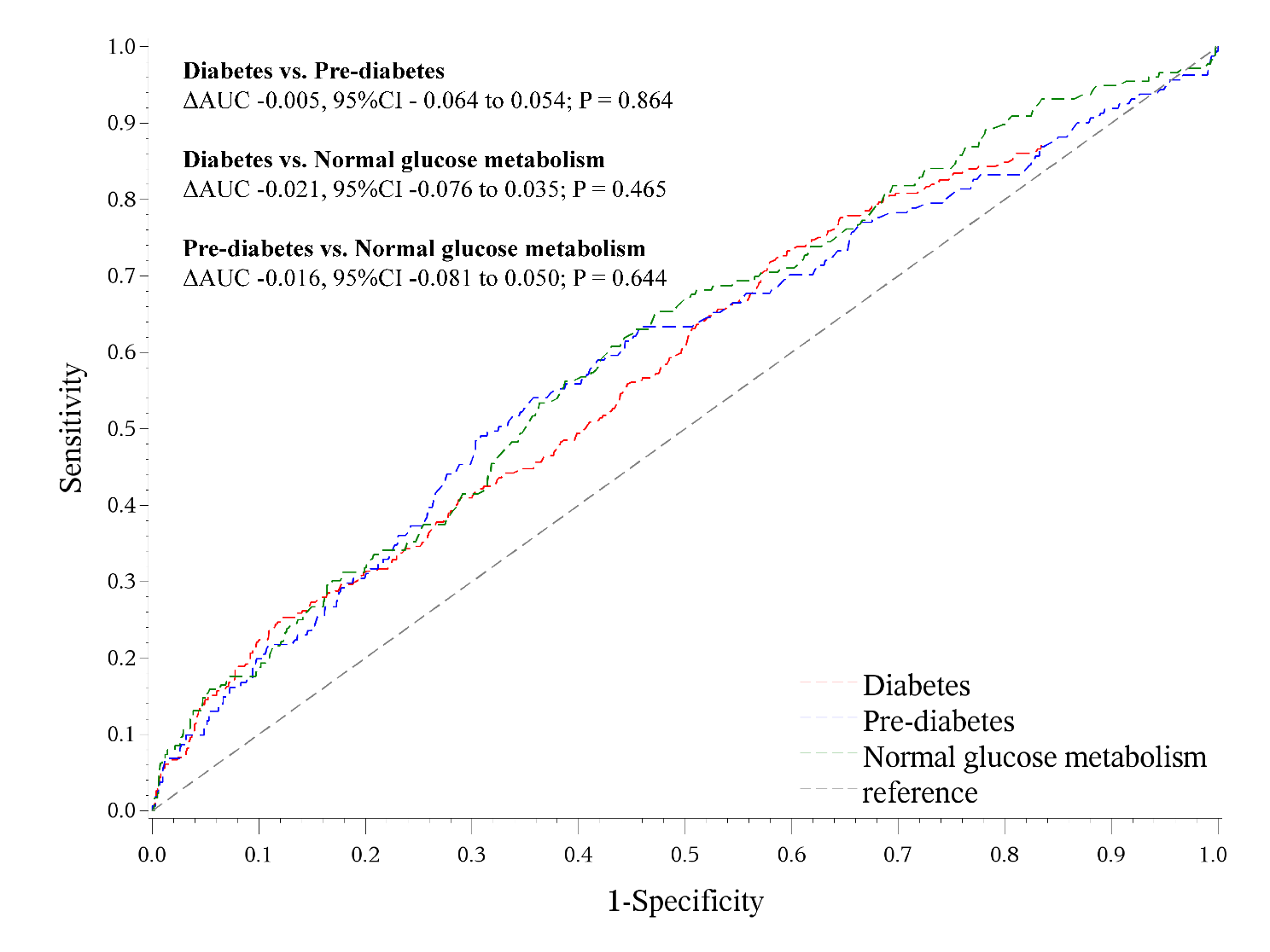
**

**Supplementary Figure 3 Kaplan-Meier curves for secondary outcomes according to ABG levels in patients with different glucose metabolism status.** **(A) diabetes; (B) pre-diabetes; (C) normal glucose regulation.** ABG, admission blood glucose; MACCE, major adverse cardiovascular and cerebrovascular event.


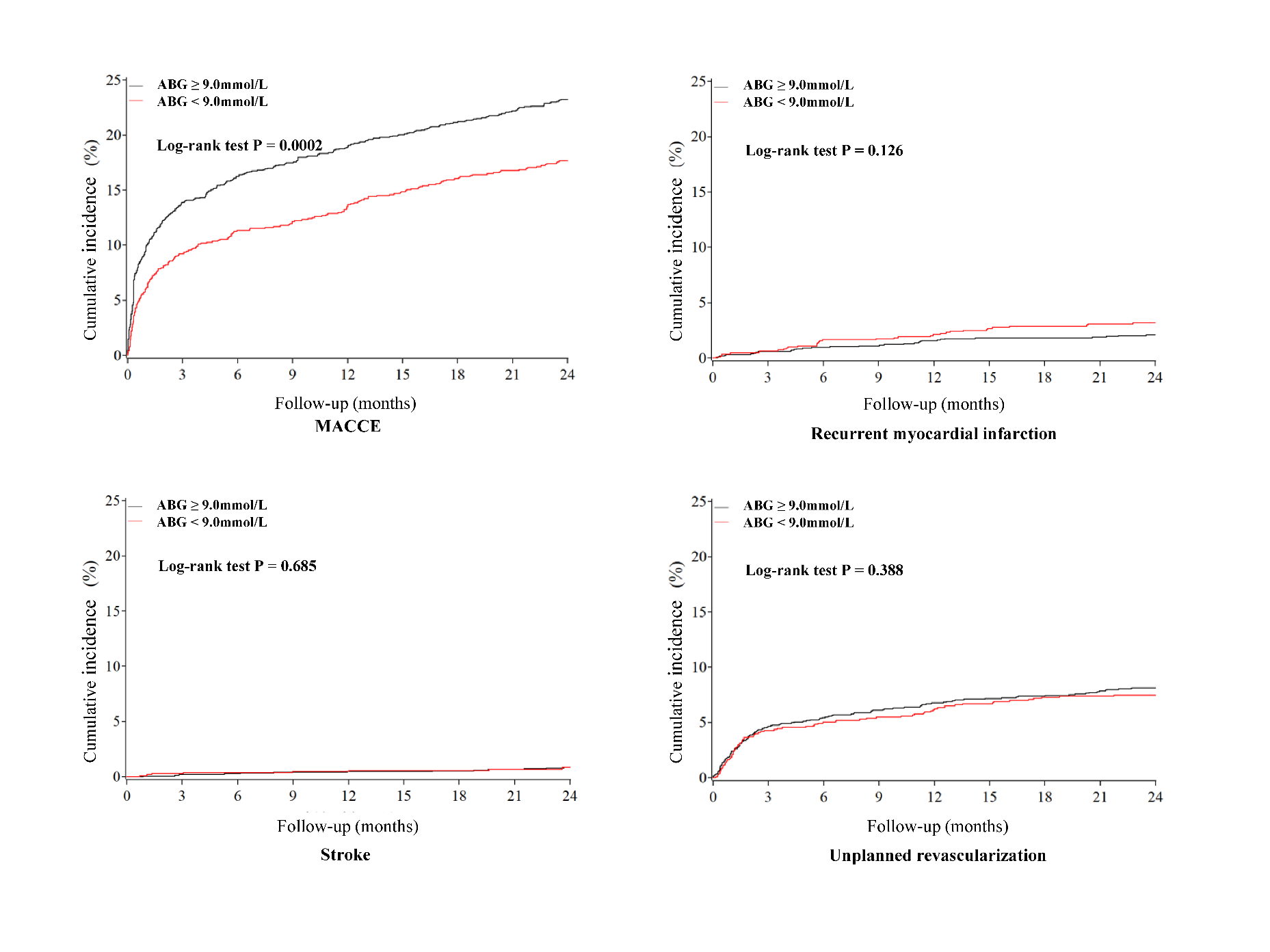


**(A)**

**
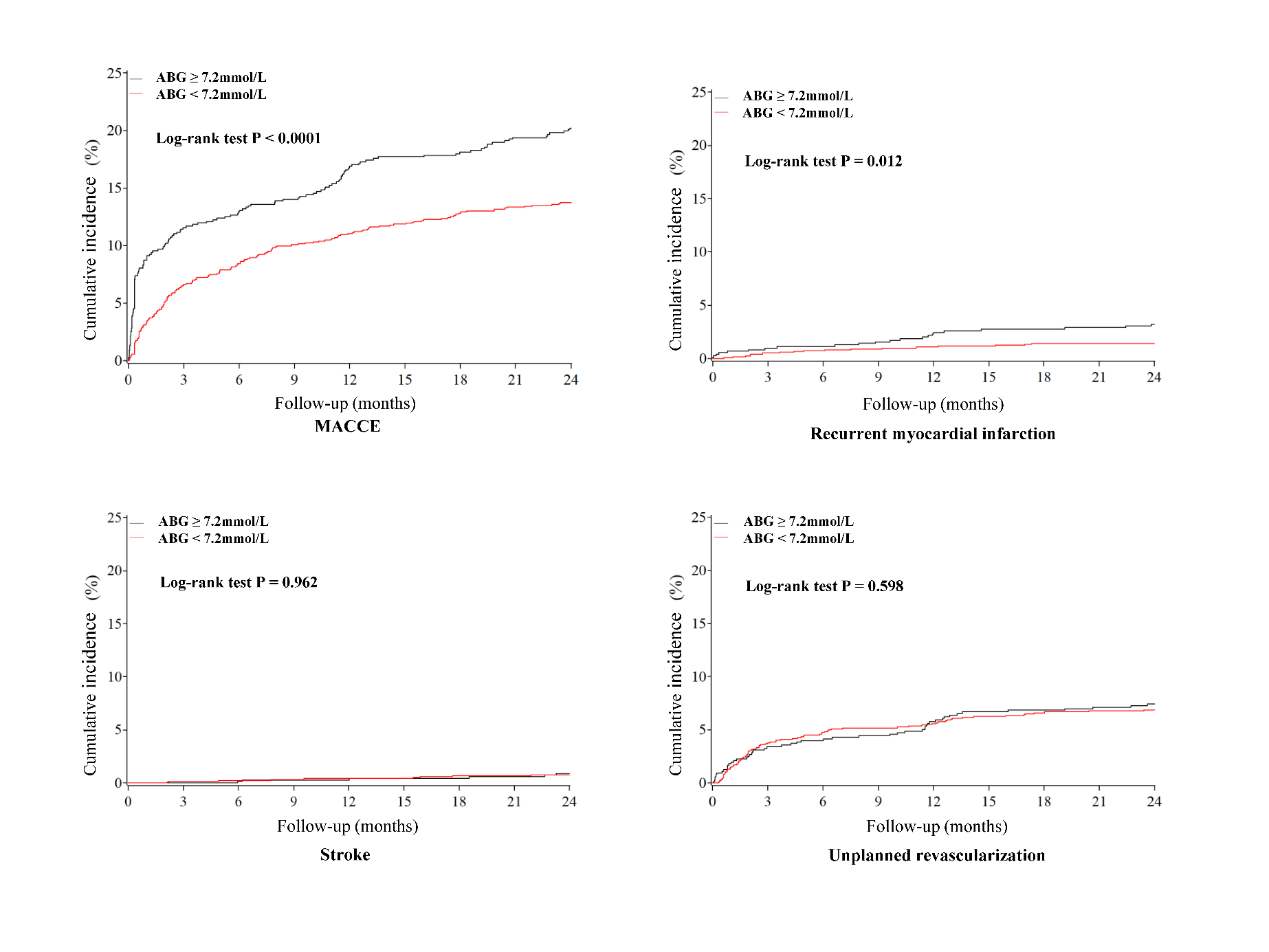
**

**(B)**

**
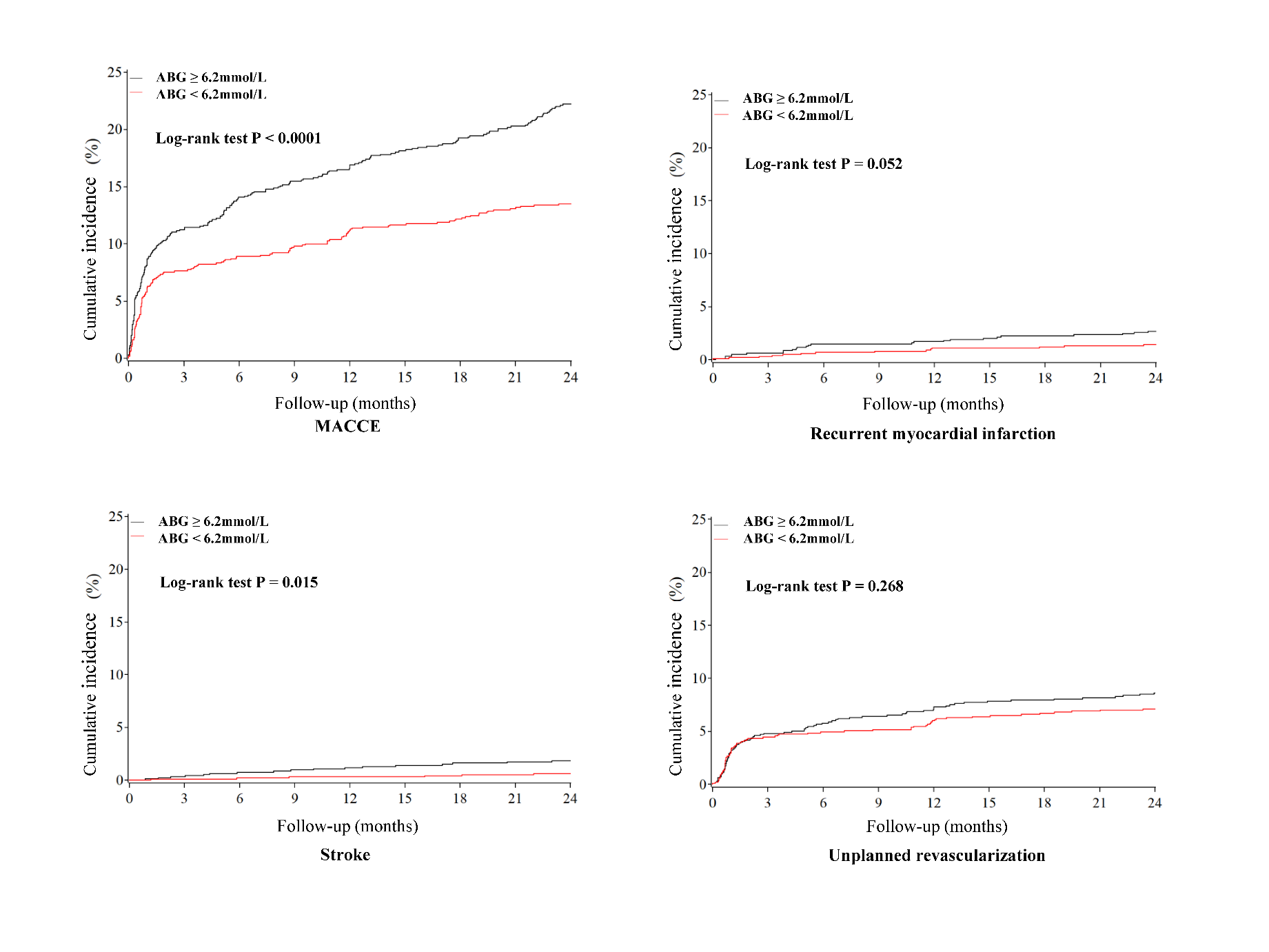
**

**(C)**
